# Genomics‐Driven Discovery of a Novel Glutarimide Antibiotic from *Burkholderia gladioli* Reveals an Unusual Polyketide Synthase Chain Release Mechanism

**DOI:** 10.1002/anie.202009007

**Published:** 2020-10-26

**Authors:** Ioanna T. Nakou, Matthew Jenner, Yousef Dashti, Isolda Romero‐Canelón, Joleen Masschelein, Eshwar Mahenthiralingam, Gregory L. Challis

**Affiliations:** ^1^ Department of Chemistry University of Warwick Coventry CV4 7AL UK; ^2^ Warwick Integrative Synthetic Biology Centre University of Warwick Coventry CV4 7AL UK; ^3^ Institute of Clinical Sciences School of Pharmacy University of Birmingham Birmingham B15 2TT UK; ^4^ Organisms and Environment Division Cardiff School of Biosciences Cardiff University Cardiff CF10 3AT UK; ^5^ Department of Biochemistry and Molecular Biology ARC Centre of Excellence for Innovations in Peptide and Protein Science Monash University Victoria 3800 Australia; ^6^ Current Address: The Centre for Bacterial Cell Biology, Biosciences Institute Medical School Newcastle University Newcastle upon Tyne NE2 4AX UK; ^7^ Current Address: Laboratory for Biomolecular Discovery &, Engineering VIB-KU Leuven Center for Microbiology Department of Biology KU Leuven 3001 Leuven Belgium

**Keywords:** anticancer agents, biosynthesis, enzymology, genome mining, natural products

## Abstract

A gene cluster encoding a cryptic trans‐acyl transferase polyketide synthase (PKS) was identified in the genomes of *Burkholderia gladioli* BCC0238 and BCC1622, both isolated from the lungs of cystic fibrosis patients. Bioinfomatics analyses indicated the PKS assembles a novel member of the glutarimide class of antibiotics, hitherto only isolated from *Streptomyces* species. Screening of a range of growth parameters led to the identification of gladiostatin, the metabolic product of the PKS. NMR spectroscopic analysis revealed that gladiostatin, which has promising activity against several human cancer cell lines and inhibits tumor cell migration, contains an unusual 2‐acyl‐4‐hydroxy‐3‐methylbutenolide in addition to the glutarimide pharmacophore. An AfsA‐like domain at the C‐terminus of the PKS was shown to catalyze condensation of 3‐ketothioesters with dihydroxyacetone phosphate, thus indicating it plays a key role in polyketide chain release and butenolide formation.

## Graphical abstract

Genome mining enabled the discovery of gladiostatin, a novel glutarimide antibiotic from the cystic fibrosis associated bacterium *Burkholderia gladioli*. Gladiostatin contains an unusual 2‐acyl‐3‐hydroxy‐4‐methylbutenolide and exhibits promising activity against human cancer cells. The polyketide synthase responsible for its biosynthesis employs a novel mechanism for chain release, resulting in formation of a phosphorylated butenolide intermediate.

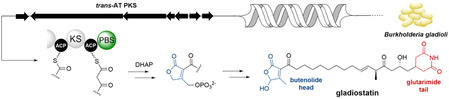

## Introduction

The constant competition between microbes and their environment has driven the evolution of specialised metabolite production in bacteria, enabling rapid ecological adaptation.^[1]^ Such metabolites frequently find important applications in medicine, as antibiotics, anticancer agents and immune modulators, and agriculture, as insecticides, herbicides and fungicides. Gram‐negative bacteria belonging to the *Burkholderia* genus produce a wide array of bioactive specialised metabolites, including the respiratory toxin bongkrekic acid, anti‐proliferative agents such as thailanstatin and spliceostatin, and the antibiotics enacyloxin IIa and gladiolin.[^2^, ^3^, ^4^, ^5^, ^6^] Despite their structural complexity and diversity, these molecules are biosynthesised from simple building blocks by modular polyketide synthase (PKS) and non‐ribosomal peptide synthetase (NRPS) assembly lines, often harbouring non‐canonical characteristics.[^7^, ^8^] Recently, we have shown that the opportunistic pathogen *Burkholderia gladioli* BCC0238, isolated from the lung of a cystic fibrosis (CF) patient, produces a range of specialised metabolites, dependent on the carbon source. When glycerol is used as the carbon source, this strain produces gladiolin, a novel macrolide with promising activity against *Mycobacterium tuberculosis*, and the swarming inhibitor icosalide A1, which was originally isolated from a filamentous fungus, but has subsequently been shown to originate from a *Burkholderia* symbiont.[^6^, ^9^, ^10^] Switching to a mixture of glycerol and ribose as carbon sources, induces the production of bolagladins A and B, novel lipodepsipeptides containing a unique citrate‐primed fatty acid and an unusual dehydro‐β‐alanine residue.^[11]^

Although several specialised metabolites and their associated biosynthetic gene clusters (BGCs) have already been identified in *B. gladioli* BCC0238, analysis of the complete genome sequence of this strain indicated that it contains numerous cryptic BGCs, the metabolic products of which are currently unknown.^[6]^ Here we report the discovery of gladiostatin, a novel member of the glutarimide class of polyketide antibiotics, as the metabolic product of a cryptic trans‐acyl transferase (trans‐AT) PKS‐encoding BGC in *B. gladioli* BCC0238. This unusual metabolite, which contains a rare 2‐acyl‐4‐hydroxy‐3‐methylbutenolide in addition to the 2, 6‐piperidinedione common to all glutarimides, is active against yeast and has promising activity against several human cancer cell lines. Gene disruption experiments confirmed that the BGC directs the biosynthesis of gladiostatin, and chain release from the PKS was reconstituted in vitro, providing insights into the mechanism for formation of the unusual butenolide moiety. These experiments enabled us to propose a biosynthetic pathway for gladiostatin, the first glutarimide to be isolated from Gram‐negative bacteria, illuminating the role played by horizontal gene transfer in trans‐AT PKS evolution.

## Results and Discussion

### Genome Mining Identifies a Cryptic trans‐AT PKS Predicted to Assemble a Novel Glutarimide

**Figure 1:**
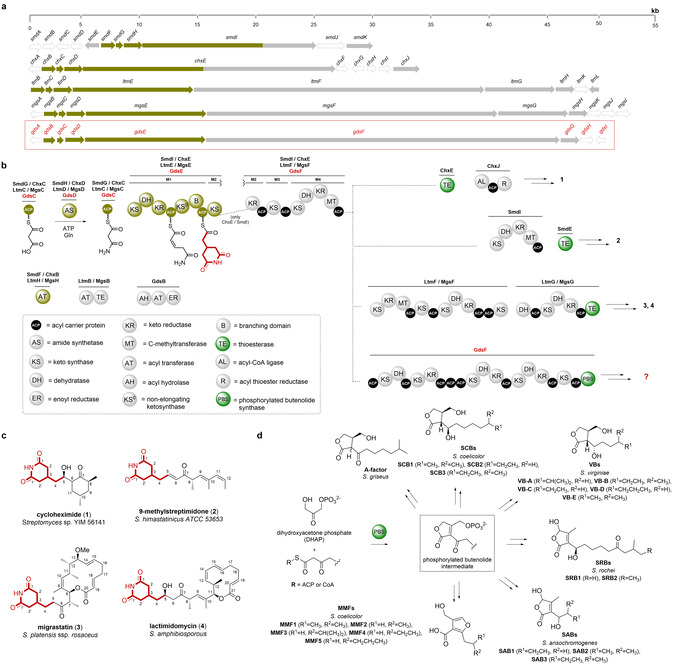
Comparison of *Streptomyces* glutarimide biosynthetic gene clusters and polyketide synthases they encode with that encoded by the cryptic *B. gladioli* gene cluster and role played by AfsA‐like phosphorylated butenolide synthases (PBSs) in *Streptomyces* signalling molecule biosynthesis. a) Comparison of biosynthetic gene clusters (BGCs) that direct glutarimide biosynthesis in *Streptomyces* species (9‐methylstreptimidone (*smd*), cycloheximide (*chx*), lactimidomycin (*ltm*) and migrastatin (*mgs*)) with the cryptic *B. gladioli* BCC0238/BCC1622 gene cluster (highlighted by the red box). The genes (or regions thereof) encoding the machinery responsible for assembling the common 2‐(2,6‐dioxopiperidin‐4‐yl)acetyl thioester biosynthetic intermediate are highlighted in gold. b) Comparison of the 9‐methylstreptimidone, cycloheximide, lactimidomycin and migrastatin PKS architectures with the PKS encoded by the cryptic *B. gladioli* BGC. Domains highlighted in dark yellow are involved in constructing the common 2‐(2,6‐dioxopiperidin‐4‐yl)acetyl thioester intermediate. Domains responsible for chain release (TE and PBS) are highlighted in green. Abbreviations for protein domains are displayed in the dashed box. c) Structures of glutarimide antibiotics produced by *Streptomyces* species. d) Structures of *Streptomyces* signalling molecules known or proposed to originate from a phosphorylated butenolide intermediate resulting from PBS‐catalysed condensation of DHAP with a 3‐ketoacyl‐ACP/CoA thioester.

Building on our previous work in *B. gladioli* BCC0238, in silico analysis of the genome sequence revealed a ≈50 kb cryptic BGC encoding a trans‐AT PKS on the second chromosomal replicon (Figure S1). Bioinformatics analyses indicated that several of the proteins encoded by this BGC are very similar to enzymes known to be involved in the biosynthesis of the glutarimide class of polyketide antibiotics in *Streptomyces* species (Figure 1 a and b), including cycloheximide (**1**),[^12^, ^13^] 9‐methylstreptimidone (**2**),[^14^, ^15^] migrastatin/iso‐migrastatin (**3**)[^16^, ^17^, ^18^] and lactimidomycin (**4**; Figure 1 c, Figure S2).[^19^, ^20^] Glutarimides have potent antifungal activity and inhibit eukaryotic translation by blocking the binding of tRNA to the E‐site of the 60S ribosomal subunit.[^21^, ^22^] They also possess promising anticancer activity and several members of the family have been reported to inhibit tumour cell‐migration.[^23^, ^24^]

Three conserved genes (*smdFGH*, *chxBCD*, *ltmHCD* and *mgsHCD* in the 9‐methylstreptimidone, cycloheximide, lactimidomycin and migrastatin BGCs, respectively) are proposed to be involved in the creation of a common malonamyl thioester starter unit for the PKSs that assembles these metabolites (Figure 1 a).[^12^, ^15^, ^16^, ^17^, ^18^, ^19^, ^20^] These genes encode: an acyltransferase (AT; SmdF/ChxB/LtmH/MgsH) that is proposed to malonylate an acyl carrier protein (ACP; SmdG/ChxC/LtmC/MgsC); and an asparagine synthetase homologue (SmdH/ChxD/LtmD/MgsD), which is hypothesised to convert the resulting malonyl thioester to a malonamyl thioester. The first module in each PKS (located in the SmdI, ChxE, LtmE and MgsE subunits) contains seven conserved domains that are proposed to elaborate the malonamyl starter unit into a common 2‐(2,6‐dioxopiperidin‐4‐yl)acetyl thioester intermediate (Figure 1 b).^[25]^

The *gdsB*, *gdsC* and *gdsD* genes in the cryptic *B. gladioli* BGC encode homologues of the three proteins hypothesised to create the malonamyl thioester starter unit (Figure 1 b). Moreover, the first module of the PKS encoded by this cluster (situated in the GdsE subunit) has an identical seven‐domain architecture to the corresponding modules of the PKSs that assemble the *Streptomyces* glutarimides. However, the domain architecture of subsequent modules in the *B. gladioli* PKS differs significantly from the *Streptomyces* glutarimide assembly lines and a unique AfsA‐like domain (pfam03756—identified by a conserved domain search) is appended to the C‐terminus of the final PKS module (Figure 1 b). AfsA catalyses the condensation of dihydroxyacetone phosphate (DHAP) with a β‐keto thioester to form a phosphorylated butenolide intermediate in the biosynthesis of A‐factor, a signalling molecule that controls morphological differentiation and antibiotic production in *Streptomyces griseus* (Figure 1 d).^[26]^ AfsA homologues are proposed to catalyse analogous reactions in the biosynthesis of other γ‐butyrolactones (GBLs), such as the *Streptomyces coelicolor* butyrolactones and the *Streptomyces virginiae* butanolides (Figure 1 d).[^27^, ^28^] Members of this enzyme family are also involved in the biosynthesis of other classes of *Streptomyces* signalling molecules, such as 2‐alkyl‐4‐hydroxymethylfuran‐3‐carboxylic acids (AHFCAs), typified by the methylenomycin furans, and 2‐alkyl‐4‐hydroxy‐3‐methylbutenolides (AHMBs), exemplified by the *Streptomyces rochei* butenolides and the *Streptomyces ansachromogenes* butenolides (Figure 1 d).[^29^, ^30^, ^31^] Incorporation experiments with stereospecifically ^13^C‐labelled glycerols indicate that an analogous phosphorylated butenolide to that formed by AfsA in A‐factor biosynthesis is an intermediate in the biosynthesis of the methylenomycin furans.^[32]^ This suggests that the AfsA family of enzymes involved in GBL, AHFCA and AHMB assembly all produce analogous phosphorylated butenolides that are diversified by subsequent biosynthetic enzymes (Figure 1 d).

PKSs typically have a thioesterase (TE) domain appended to the C‐terminus of the last module, which catalyses release of the fully assembled polyketide chain via hydrolysis or macrocyclisation, but several other chain release mechanisms are known.[^33^, ^34^, ^35^, ^36^] Based on the role played by AfsA family enzymes in *Streptomyces* signalling molecule biosynthesis, we hypothesised that the AfsA‐like domain appended to the C‐terminus of the last module in the cryptic *B. gladioli* PKS releases the fully assembled polyketide chain by condensing it with DHAP to form a phosphorylated butenolide (Figure 1 d). Accordingly, we designated this new type of chain release enzyme a phosphorylated butenolide synthase (PBS) domain. Overall, our in silico analyses indicated that the cryptic *B. gladioli* PKS assembles a novel glutarimide‐containing polyketide with significant structural differences to the glutarimide antibiotics assembled by *Streptomyces* species.

### Isolation and Structure Elucidation of Metabolic Products of the Cryptic PKS

We originally identified the BGC encoding the cryptic trans‐AT PKS in *B. gladioli* BCC0238. However, due to difficulties with creating in‐frame deletions in this strain, we searched the genomes of other *B. gladioli* strains to see if they contain this BGC. Another CF isolate, *B. gladioli* BCC1622, which is more amenable to genetic manipulation,^[11]^ was also found to contain the cluster. UHPLC‐ESI‐Q‐TOF‐MS analysis of an ethyl acetate extract from a culture of *B. gladioli* BCC1622, grown for 3 days on a minimal agar medium containing glycerol as the sole carbon source, identified a metabolite with the molecular formula C_23_H_37_NO_5_ (Figure S3).

**Figure 2:**
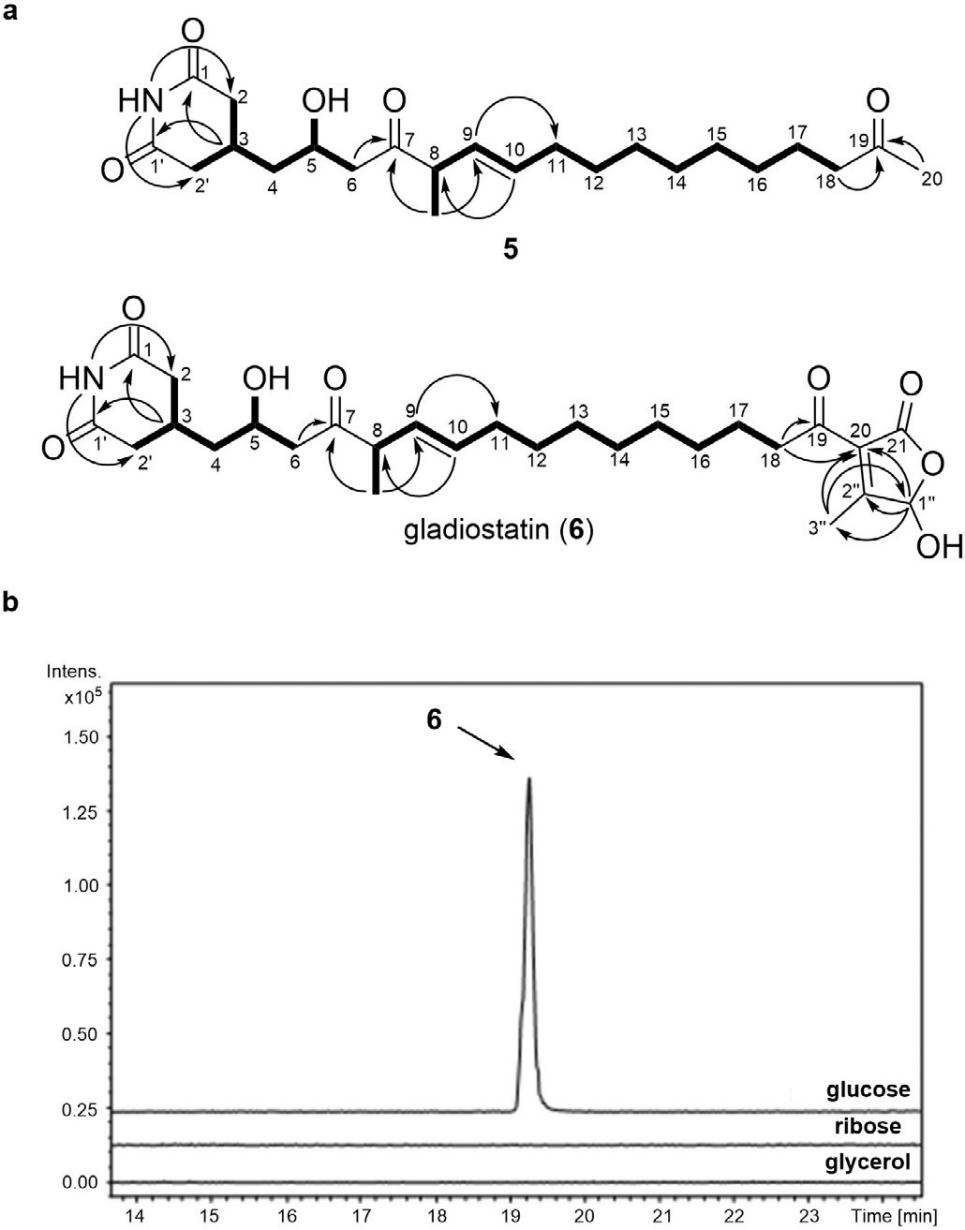
Identification and structure elucidation of metabolic products of the cryptic trans‐AT PKS in *B. gladioli* BCC1622. a) Structure elucidation of degradation product (**5**) (*top*) and gladiostatin (**6**) (*bottom*). COSY and key HMBC correlations observed for each compound are indicated by bold lines and arrows, respectively. b) Extracted ion chromatograms at *m*/*z*=506.27±0.02, (corresponding to the [*M*+H]^+^ ion of gladiostatin) from UHPLC‐ESI‐Q‐TOF‐MS analysis of ethyl acetate extracts from *B. gladioli* BCC1622 grown on a minimal medium containing different carbon sources.

The planar structure of this metabolite (**5**) was elucidated using ^1^H, ^13^C, COSY, HSQC and HMBC NMR experiments (Figure 2 a, Figure S4–S8, Table S1). HMBC correlations between H‐3 and C‐1/C‐1′ and the exchangeable NH proton and C‐2/C‐2′ confirmed the presence of a 2, 6‐piperidinedione, and the chemical shift values for this moiety were in good agreement with the literature.[^18^, ^19^, ^37^, ^38^] Two networks of COSY correlations established the structures of the C‐2/C‐2′ to C‐6 and C‐8 to C‐18 regions of the molecule, and HMBC correlations further confirmed the locations of the C‐8 Me group and the C‐9/C‐10 double bond. HMBC correlations also showed that C‐6 and C‐8 are connected via a keto group and that a methyl ketone is attached to C‐18. Based on a ^3^ *J*_HH_ coupling constant of 15 Hz, the C‐9/C‐10 double bond was assigned the *E* configuration.

Although compound **5** contains the anticipated 2, 6‐piperidinedione, it lacks the butenolide predicted to be installed by the PBS domain (Figure 1 d). We thus postulated that this compound results from degradation of the true metabolic product of the BGC. A range of carbon sources (glycerol, glucose, ribose) and growth periods were explored to investigate whether other metabolic products of the BGC could be identified. UHPLC‐ESI‐Q‐TOF‐MS analysis of an ethyl acetate extract from a 24 hour culture on a minimal medium containing glucose as the sole carbon source identified a new metabolite with the molecular formula C_27_H_39_NO_8_ (Figure 2 b and Figure S9). A time course showed that production of this metabolite peaked at 20 hours and fell off rapidly over more protracted growth periods (Figure S10). After 55 hours, small amounts of compound **5** could be detected, consistent with this being a degradation product of the new metabolite.

Comparison of the molecular formulae for compound **5** and the newly identified metabolite showed the latter contains four additional carbon atoms, and three additional hydrogen and oxygen atoms. The planar structure of the new metabolite (**6**) was elucidated using ^1^H, ^13^C, COSY, HSQC and HMBC NMR experiments (Figure 2 a, Figure S11–S15, Table S2). The ^13^C NMR spectrum of **6** lacked the signal due to methyl ketone in **5** and contained additional signals assigned to a carbonyl group (C‐21), two fully substituted alkene carbons (C‐20 and C‐2′′), a methyl group (C‐3′′) and a hemiacetal (C‐1′′). HMBC correlations between the C‐3′′ protons and C‐1′′/C‐20 and the C‐1′′ proton and C‐2′′/C‐21 led us to propose that this molecule contains a 2‐subsituted 4‐hydroxy‐3‐methylbutenolide. The NMR data for this moiety are similar to those reported for the AHMBs isolated from *S. rochei* and *S. ansachromogenes* (Figure 1 d).[^30^, ^31^] An HMBC correlation between the C‐18 protons and C‐20 established the connectivity between the butenolide and the rest of the structure. The juxtaposition of a 2‐acyl‐4‐hydroxy‐3‐methylbutenolide and 2, 6‐piperidinedione in this molecule, coupled with the time course data for its production, led us to conclude that this compound, which we named gladiostatin, is the true metabolic product of the cryptic glutarimide‐like BGC in *B. gladioli* BCC1622 and BCC0238. Gladiostatin (**6**) is likely degraded to **5** via conjugate addition of water to the butenolide, followed by ring opening, decarboxylation and retro‐Aldol cleavage (Figure S16).

### Biological Activity of Gladiostatin

Initially, we tested the activity of gladiostatin (**6**) against representative members of the ESKAPE panel of bacterial pathogens, *Candida albicans* and *Saccharomyces cerevisiae*. While no activity was detected against the bacterial pathogens or *C. albicans* at concentrations up to 64 μg mL^−1^, gladiostatin was found to be active against *S. cerevisiae*, with an MIC of 4 μg mL^−1^ (Table 1).

**Table 1:** Antimicrobial activity of gladiostatin (MIC=minimum inhibitory concentration).

Strain	MIC [μg mL^−1^]
Gram‐negative Bacteria	
*Klebsiella pneumonia* DSM 26371	>64
*Acinetobacter baumannii* DSM25645	>64
*Pseudomonas aeruginosa* DSM29239	>64
*Enterobacter cloacae* DSM 16690	>64
Gram‐positive Bacteria	
*Enterococcus faecium* DSM25390	>64
*Staphylococcus aureus* DSM21979	>64
Fungi	
*Candida albicans* SC 5314	>64
Yeast	
*S. cerevisiae* W303‐1 a	4

Prompted by reports that several glutarimides have anti‐tumour activity,[^23^, ^24^] we investigated the activity of gladiostatin (**6**) against a range of cancer cell lines (Table 2). It was found to be active against ovarian, pancreatic and colon cancer cell lines (Table 2). These values are in the same range as those reported for cycloheximide (**1**), migrastatin (**3**) and lactimidomycin (**4**) against various other cell lines (Table S6).[^17^, ^19^, ^38^] Interestingly, gladiostatin (**6**) was found to be inactive against the A549 lung cancer cell line, indicating it may exhibit some selectivity.

**Table 2:** Anticancer activity of gladiostatin.

	Gladiostatin (**6**)
Cancer cell line	IC_50_ [μΜ]
A2780 (ovarian)	0.24±0.03
A549 (lung)	no activity
MiaPaca2 (pancreatic)	0.57±0.06
HCTT116 P53 ‐/‐ (colon)	0.82±0.04
SKOV3 (ovarian)	0.6±0.1
PEA1 (ovarian)	1.4±0.1

**Figure 3:**
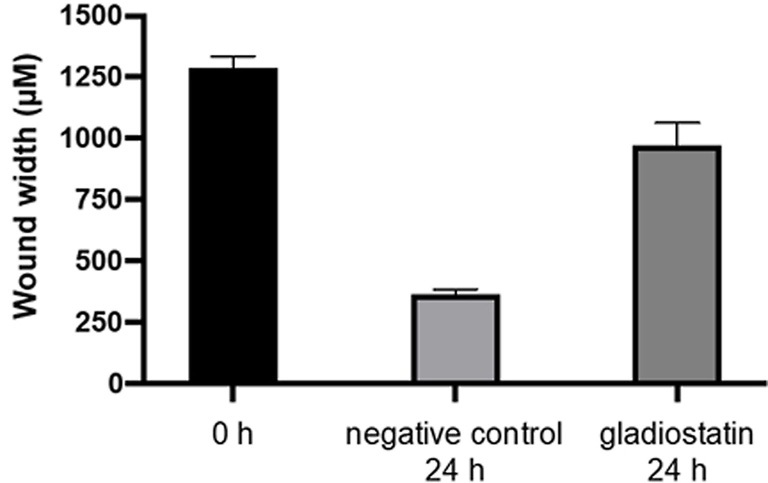
Inhibition of A2780 ovarian tumour cell migration by gladiostatin. The bar chart compares wound‐widths after 0 and 24 h in the absence of gladiostatin with the wound width after 24 h in the presence of 240 nM gladiostatin.

Some glutarimides have also been reported to inhibit tumour cell migration.[^23^, ^24^] Thus, we used a wound‐healing assay to test whether gladiostatin (**6**) can inhibit the migration of A2780 ovarian cancer cells. Strong suppression of cell migration was observed after 24 h exposure to 240 nM gladiostatin (Figure 3, Figure S17).

### Proposed Pathway for Gladiostatin Biosynthesis.

**Figure 4:**
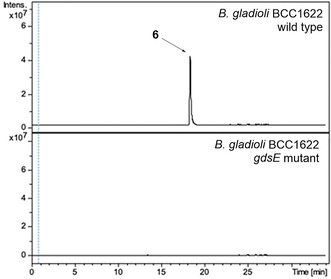
Insertional inactivation of *gdsE* abolishes gladiostatin production in *B. gladioli* BCC1622. Extracted ion chromatograms at *m*/*z* 506.27±0.02 (corresponding to [*M*+H]^+^ for gladiostatin) from UHPLC‐ESI‐Q‐TOF‐MS analysis of ethyl acetate extracts of *B. gladioli* BCC1622 (top) and the *gdsE* mutant (bottom).

To establish that the *gds* cluster directs the biosynthesis of gladiostatin (**6**), *gdsE*, which encodes the first polyketide synthase subunit, was disrupted in *B. gladioli* BCC1622 by insertional mutagenesis. LC‐MS comparison of extracts from the wild type and mutant strains confirmed that gladiostatin (**6**) production is abolished in the mutant (Figure 4).

**Figure 5:**
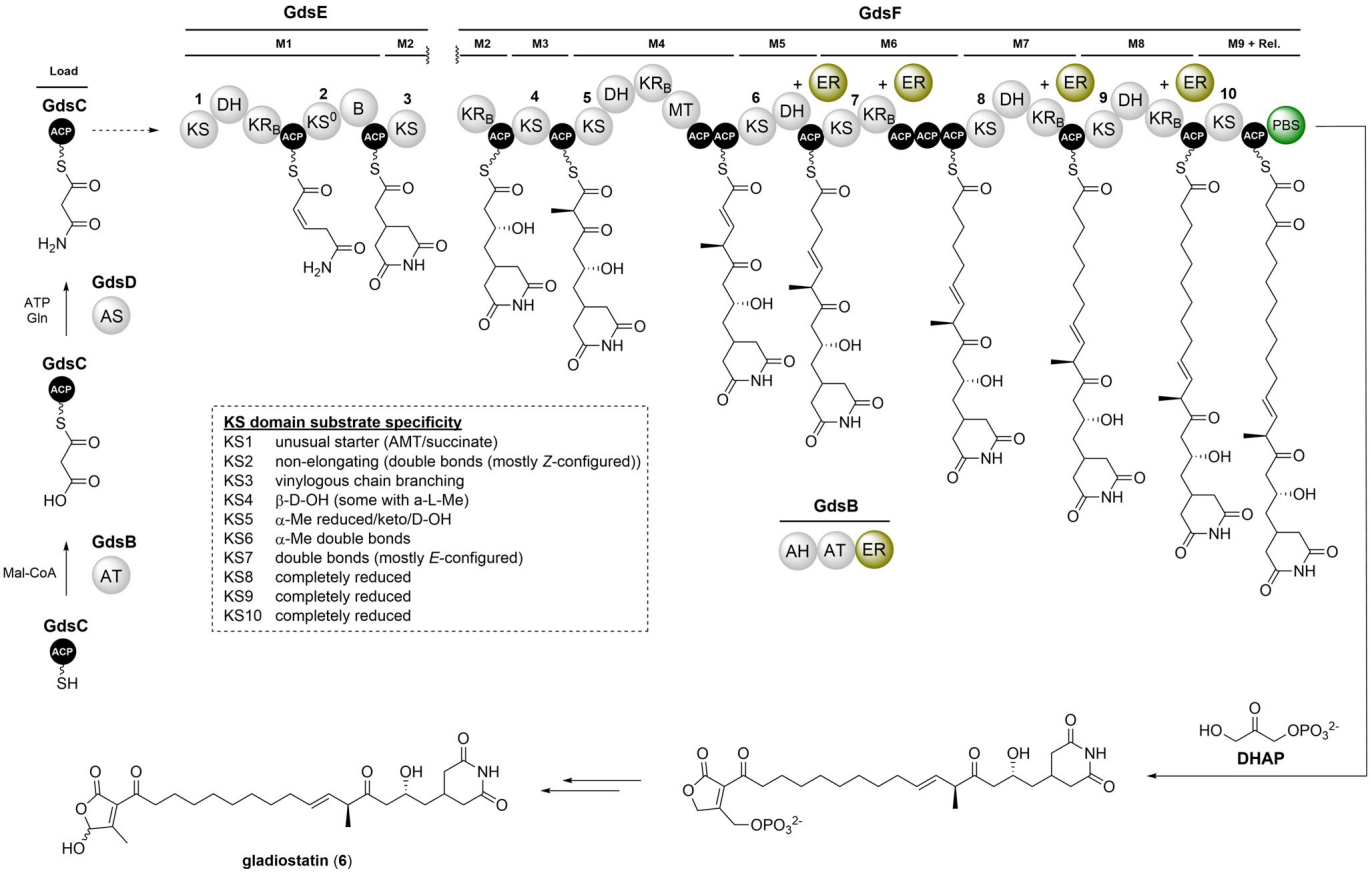
Proposed pathway for gladiostatin biosynthesis. Domain and module organization of the gladiostatin trans‐AT PKS showing the proposed structure of each ACP‐bound thioester intermediate. KS domains have been numbered sequentially, and the TransATor^[42]^ predictions for their acyl‐ACP substrates are shown in the dashed box. Modules are labelled M1, M2 etc. The A/B subscripts denote the predicted stereospecificity of KR domains. The trans‐acting ER domain (gold) of GdsB is predicted to reduce enoyl thioesters attached to the ACP domains in modules 5, 6, 7 and 8. Domain abbreviations are as defined in Figure 1. The putative functions of the proteins encoded by the gladiostatin biosynthetic gene cluster are listed in Table S3. The stereochemistry of C‐5 and C‐8 is hypothesised to be the same as the corresponding stereocentres in lactimidomycin. Comparative sequence analysis of the module 2 KR domain predicts that it produces an R‐configured alcohol (Table S5), consistent with this hypothesis.

The early stages of gladiostatin (**6**) biosynthesis appear to parallel the assembly of glutarimides in *Streptomyces* species. GdsB and GdsD are proposed to create a GdsC‐bound malonamyl thioester starter unit, which is elaborated to a 2‐(2,6‐dioxopiperidin‐4‐yl)acetyl thioester intermediate by the first PKS module (Figure 5). Modules 2, 3 and 4 have identical domain architectures in all glutarimide PKSs (Figure 1 b). In the *Streptomyces* assembly lines, a single MT domain in module 4 seems to be responsible for methylating the α‐carbon of the β‐keto‐ACP thioester intermediate generated by the ketosynthase (KS) domain in both modules 3 and 4 of the PKSs, installing methyl branches at C‐8 and C‐10 of the resulting glutarimides. In contrast, the MT domain in module 4 of the gladiostatin PKS appears to methylate only the β‐keto thioester intermediate attached to the module 3 ACP domain (Figure 5). Consequently, gladiostatin (**6**) has a methyl branch at C‐8, but not C‐10. Additional studies are needed to understand how PKSs with seemingly identical architectures are able to produce the distinct C‐methylation patterns observed in gladiostatin (**6**) and the *Streptomyces* glutarimides.

The domain architecture of modules 5–9 of the gladiostatin PKS diverges significantly from the corresponding modules in the *Streptomyces* glutarimide assembly lines. Indeed the C‐terminus of module 4 appears to be a branch point in all glutarimide‐producing PKSs (Figure 1 b), and could be a recombination hotspot. Based on comparisons between the domain architecture and the predicted biosynthetic intermediates, module 5 of the gladiostatin PKS appears to lack both a ketoreductase (KR) domain (which is required for installation of the β‐hydroxy group) and an enoyl reductase (ER) domain (required to saturate the double bond introduced by the dehydratase (DH) domain). Similarly, module 6 lacks a DH domain and modules 6, 7 and 8 lack ER domains, suggesting a high degree of non‐linear programming in the gladiostatin PKS, which is a common feature of glutarimide assembly lines (Figure 1).^[39]^

While it is unclear which domains are responsible for the keto reduction and dehydration reactions in modules 5 and 6, respectively, in silico analysis of the gladiostatin BGC identified two genes encoding putative reductases that could function as trans‐acting ERs in modules 5–8. The *gdsB* gene encodes a tri‐domain protein with acyl hydrolase (AH) and AT domains fused to a flavin‐dependent ER domain and *gdsH* encodes an NAD(P)H‐dependent oxidoreductase. We therefore propose that one, or both, of these reductases catalyse enoyl reduction in modules 5, 6, 7 and 8 of the PKS. A recently reported co‐evolutionary categorisation of trans‐AT PKS ACP domains supports this hypothesis, indicating that the module 6, 7 and 8 ACP domains in the gladiostatin PKS clade with ACP domains in modules with a similar architecture from other assembly lines (i.e. KS‐DH‐KR‐ACP and a trans‐acting ER).^[40]^ Moreover, the TransATor software predicts that the KS domains in modules 7, 8 and 9 are selective for an α,β‐saturated thioester intermediate.^[41]^ However, this software also predicts that the module 6 KS domain prefers a α,β‐unsaturated thioester, highlighting potential limitations of such predictive bioinformatics analyses (Figure 5, Figure S18 and S19; Table S4 and S7).

The most striking difference between the gladiostatin PKS and the other glutarimide assembly lines is the mechanism for polyketide chain release. All of the *Streptomyces* glutarimide PKSs are proposed to use TE domains that catalyse hydrolysis or macrolactonisation. In contrast, the gladiostatin PKS appears to employ a PBS domain to catalyse condensation of the β‐ketothioester attached to the last module of the PKS with DHAP. The resulting phosphorylated butenolide is proposed to undergo rearrangement and dephosphorylation to afford a 2‐acyl‐4‐hydroxy‐3‐methylbutenolide (Figure 5). This hypothesis is consistent with the observed increase in gladiostation production levels when glucose is used as the carbon source, because DHAP is an intermediate in glycolysis. To our knowledge, there is no precedent for PKS chain release by a PBS domain. However, PBSs are known to catalyse the condensation of DHAP with a range of β‐ketothioesters in the biosynthesis of several distinct classes of *Streptomyces* signalling molecule (Figure 1 d). These include the SRBs and the SABs, which contain a similar butenolide moiety to gladiostatin (**6**). In addition to the PBS (SabA), a phosphatase (SabP) and a ketoreductase (SabD) are known to be required for SAB biosynthesis, but the mechanism for elaboration of the putative phosphorylated butenolide produced by SabA to the 2‐akyl‐4‐hydroxy‐3‐methylbutenolide remains to be elucidated. The *gdsA* and *gdsG* genes in the gladiostatin BGC encode putative phosphatases that could play a similar role to SabP in the biosynthesis of the SABs (Table S3).

### In vitro Reconstitution of Chain Release from the Gladiostatin PKS

To validate the proposed role of the PBS domain in gladiostatin (**6**) biosynthesis, we investigated its catalytic activity using simplified mimics of the fully assembled polyketide chain. A synthetic gene encoding the C‐terminal ACP and PBS domains of GdsF was used to overproduce the ACP‐PBS di‐domain in *E. coli* as an N‐terminal His_8_‐fusion, which was purified to homogeneity using immobilised metal‐ion affinity chromatography. The identity of the purified protein was confirmed by ESI‐Q‐TOF‐MS analysis (Figure S20).

The ability of the PBS domain to offload a 3‐ketothioester from the PKS was examined by loading a 3‐ketobutyryl mimic of the fully assembled polyketide chain onto the *apo*‐ACP domain using the promiscuous phosphopantetheinyl transferase Sfp (Figure S21). The mass of the protein decreased by 86 Da when DHAP was added (Figure S21), consistent with cleavage of the 3‐ketobutyryl group from the ACP domain.

**Figure 6:**
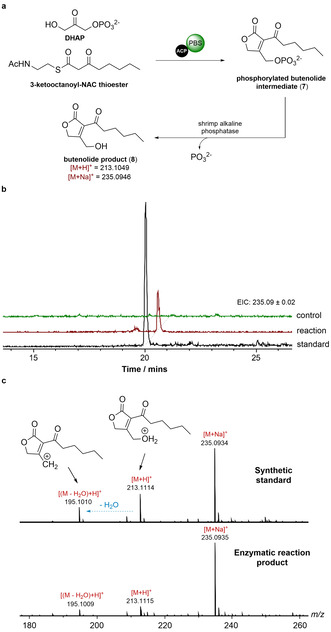
In vitro reconstitution of chain release by the PBS domain. a) Condensation of DHAP and the NAC thioester of 3‐ketooctanoate by the purified recombinant GdsF ACP‐PBS di‐domain and dephosphorylation of the resulting phosphorylated butenolide (**7**) using shrimp alkaline phosphatase. b) Extracted‐ion chromatograms at *m*/*z=*235.0925 (corresponding to [*M*+Na]^+^ for butenolide **8**) from UHPLC‐ESI‐Q‐TOF‐MS comparisons of the enzymatic reaction cascade and a synthetic standard of **8**. The ACP‐PBS di‐domain was omitted from the control reaction. c) Comparison of MS/MS spectra for the synthetic standard of butenolide **8** ([*M*+H]^+^=213.11) and the product of the enzymatic reaction cascade.

Incubation of the *apo*‐ACP‐PBS di‐domain with the N‐acetylcystetamine (NAC) thioester of 3‐ketooctanoate and DHAP, followed by treatment with shrimp alkaline phosphatase yielded a product, absent from the negative control, that gave rise to ions with *m*/*z*=213.11 and *m*/*z*=235.09 (corresponding to the [*M*+H]^+^ and [*M*+Na]^+^, respectively, of the 2‐acyl‐3‐hydroxymethyl butenolide (**8**)) in UHPLC‐ESI‐Q‐TOF‐MS analyses (Figure 6). A synthetic standard of **8** had the same retention time and MS/MS fragmentation pattern as the product of the enzymatic reaction (Figure 6).

## Conclusion

The discovery of gladiostatin (**6**) as the product of a cryptic trans‐AT PKS assembly line in *B. gladioli* BCC0238 and BCC1622 further expands the already rich specialised metabolic repertoire of these and related CF isolates.[^6^, ^9^, ^11^] As the first glutarimide antibiotic to be isolated from a Gram‐negative bacterium, gladiostatin differs significantly from other members of this family, all of which are produced by *Streptomyces* species. While some of these structural differences (such as the fully saturated C‐11 to C‐18 chain and the 2‐acyl‐4‐hydroxy‐3‐methylbutenolide) are reflected by alterations in PKS architecture, others (e.g. the lack of a C‐10 methyl group) are not. The discovery of gladiostatin thus offers a golden opportunity to develop a better understanding of the role played by horizontal gene transfer in trans‐AT PKS evolution, which could facilitate biosynthetic engineering approaches to polyketide structural diversification.

In vitro characterisation of the AfsA‐like PBS domain appended to the C‐terminus of the gladiostatin PKS shows that it releases 3‐keto thioesters from the upstream ACP domain by condensing them with DHAP. This constitutes a new mechanism for polyketide chain release, which could prove to be a valuable addition to the synthetic biology toolbox. The phosphorylated butenolide product of the PBS domain is analogous to the intermediate in A‐factor biosynthesis produced by AfsA. The same kind of intermediate has been shown to be formed by MmfL (another AfsA homologue) in the biosynthesis of the methylenomycin furans.^[42]^ Thus, it appears that phosphorylated 2‐acyl‐3‐hydroxymethylbutenolide intermediates are involved in the biosynthesis of structurally diverse natural products, including GBLs, AHFCAs, AHMBs and gladiostatin. However, with the exception of GBLs, further work is needed to understand the mechanisms by which these intermediates get elaborated into the final metabolic products.

## Conflict of interest

The authors declare no conflict of interest.

## Supporting information

As a service to our authors and readers, this journal provides supporting information supplied by the authors. Such materials are peer reviewed and may be re‐organized for online delivery, but are not copy‐edited or typeset. Technical support issues arising from supporting information (other than missing files) should be addressed to the authors.

Supplementary
