# Supplementary material for: Genomics‐Driven Discovery of a Novel Glutarimide Antibiotic from Burkholderia gladioli Reveals an Unusual Polyketide Synthase Chain Release Mechanism
Source: Angew Chem Int Ed Engl. 2020 Oct 26;59(51):23145–53. doi: 10.1002/anie.202009007 (PMC7756379; doi:10.1002/anie.202009007)
Supplement: Supplementary file 1 — Supplementary [file ANIE-59-23145-s001.pdf]

Angewandte
Eine Zeitschrift der Gesellschaft Deutscher Chemiker
Chemie

Supporting Information

Genomics-Driven Discovery of a Novel Glutarimide Antibiotic from *Burkholderia gladioli* Reveals an Unusual Polyketide Synthase Chain Release Mechanism

*Ioanna T. Nakou+, Matthew Jenner+, Yousef Dashti+, Isolda Romero-Canelón, Joleen Masschelein, Eshwar Mahenthiralingam, and Gregory L. Challis**

anie_202009007_sm_miscellaneous_information.pdf

1. Materials and Methods

1.1 Strains and culture conditions

The strains used in this study were *B. gladioli* BCC0238 and *B. gladioli* BCC1622, which were drawn from previous studies[1,2], and had been accurately identified to the species level by either MLST[3] or *recA*[4] sequence analysis. The genome sequences of these strains are accessible through the NCBI using the accession numbers detailed below. The base range spanning the gladiostatin (*gds*) biosynthetic gene cluster in each strain is given.

***B. gladioli* BCC0238**
BioSample = SAMEA3473674
BioProject = PRJEB9765
GenBank Assembly Accession = GCA_900631635
gds cluster base pair Range = 3,449,913 – 3,530,217

***B. gladioli* BCC1622**
BioSample = SAMEA4026939
BioProject = PRJEB6927
GenBank Assembly Accession = GCA_900608515
gds cluster base pair Range = 731,181 – 815,548

The strains were grown (at 30 °C) in tryptic soya medium, whereas production of metabolites was performed on a minimal salts medium with 4 g/L glucose as the carbon source, for 1 day at 30 °C[5].

1.2 Gladiostatin isolation, structure elucidation and time-course analysis

To establish the optimum conditions for gladiostatin production, a time-course analysis was performed by plating *B. gladioli* BCC1622 on Basal Salts Medium (BSM) agar, supplemented with glucose/ribose/glycerol at 30 °C and extracting using ethyl acetate every 3 hours for 4 days. The extracts were analysed using UHPLC-ESI-Q-TOF-MS.

The UHPLC-ESI-Q-TOF-MS analyses were conducted on a Dionex UltiMate 3000 UHPLC attached to a Zorbax Eclipse Plus C_{18} (100 × 2.1 mm, 1.8 µm) connected to a Bruker MaXis IMPACT mass spectrometer. Mobile phases A (water) and B (acetonitrile) were supplemented with 0.1 % formic acid. A gradient of 5% B to 100% over 45 minutes and a flow rate of 0.2 mL/min was used. The mass spectrometer was operated in positive ion mode and a range of m/z = 50-3000 was screened. Source conditions were: end plate offset at −500 V; capillary at −4500 V; nebulizer gas (N_2) at 1.6 bar; dry gas (N_2) at 8 L min^{-1}; dry temperature at 180 °C. Ion transfer conditions were: ion funnel RF at 200 Vpp; multiple RF at 200 Vpp; quadrupole low mass at 55 m/z; collision energy at 5.0 eV; collision RF at 600 Vpp; ion cooler RF at 50–350 Vpp; transfer time at 121 µs; pre-pulse storage time at 1 µs. Calibration was performed using 1 mM sodium formate through loop injection (20 µL) at the start of each run.

After establishing optimal production conditions, large-scale cultures of *B. gladioli* BCC1622 were grown on BSM agar supplemented with glucose for 20 hours. Ethyl acetate extracts of the agar were fractionated by semi-preparative HPLC on a reverse-phase C_{18} Betasil column (21.2 mm × 150 mm), monitoring absorbance at 210 nm. Mobile phases consisted of water (A) and acetonitrile (B). A gradient of 5% B to 100% B over 45 minutes was used at a flow rate of 9 mL/min.

The structure of gladiostatin was elucidated using and 1- and 2-D NMR experiments. Purified gladiostatin and the degradation product were dissolved in $CDCl_3$ and DMSO-d_6, respectively, and ^1H, ^{13}C, COSY, HSQC and HSBC spectra were recorded on a Bruker 500 MHz spectrometer equipped with a TCI cryoprobe at 25 °C. The ^1H and ^{13}C NMR signals were referenced to the residual protiated solvent at (δ_H 7.26 and δ_C 77.16 for $CHCl_3$ and δ_H 2.5 and δ_C 39.52 for DMSO).

1.3 Insertional Mutagenesis of Gladiostatin Biosynthetic Gene *gdsE*

Insertional mutagenesis was performed using the pGpΩTp suicide plasmid. A 700 bp region internal to *gdsE* was amplified using the following primers: gdsE_Bdom_For: 5’-GACTCTAGACGAGCCCGTTCGAGAAGGT-3’ and gdsE_Bdom_Rev: 5’-CTCGAATTCAGGAAGTGCGCGACCTGCT-3’ and DNA polymerase. The amplimer was digested with *Xba*I and *Eco*RI (NEB) and cloned into pGpΩTp that had been digested with the same restriction enzymes. The integrity of the resulting plasmid was confirmed by sequencing, it was introduced into *E. coli* SY327 by electroporation and subsequently mobilised into *B. gladioli* BCC1622 via triparental mating. Transconjugants were selected for using trimethoprim (150 µg/mL) and polymyxin B (600 U/mL). A single *B. gladioli* BCC1622 *gdsE* transconjugant was picked, and the desired insertion was confirmed by sequencing using the following primers: 5’-GACAACGTGCTGTCGATCGAC-3’ and 5’-GCCAGGGATGTAACGCACTG-3’.

1.4 Overproduction and purification of the GdsF His$_8$-ACP-PBS di-domain

A pET24a_pHis8-ACP-PBS construct was purchased from Epoch Life Sciences. This vector was used to transform *E. coli* TOP10 cells (Invitrogen), which were plated on LB agar containing kanamycin (50 µg/mL). Colonies were picked and grown overnight in LB medium. The plasmid was isolated from the culture using a miniprep kit (ThermoFisher Scientific), and the insert was sequenced to verify its integrity. A single colony of *E. coli* BL21(DE3) that had been transformed with the expression vector was picked and used to inoculate LB medium (10 mL) containing kanamycin (50 µg/mL). The resulting culture was incubated overnight at 37˚C and 180 rpm, then used to inoculate LB medium (1 L) containing kanamycin (50 µg/mL). The resulting culture was incubated at 37˚C and 180 rpm until the optical density of the culture at 595 nm reached 0.6, then IPTG (1 mM) was added and growth was continued overnight at 15˚C and 180 rpm.

The cells were harvested by centrifugation (4,000 x g, 15 min, 4 °C) and re-suspended in buffer (20 mM Tris-HCl, 100 mM NaCl, 20 mM Imidazole, pH 7.4) at 10 mL/L of growth medium then lysed using a Constant Systems cell disruptor. The lysate was centrifuged (37,000 x g, 30 min, 4˚C) and the resulting supernatant was loaded onto a HiTrap Chelating Column (GE Healthcare), which had been pre-loaded with 100 mM $NiSO_4$ and equilibrated in re-suspension buffer (20 mM Tris-HCl, 100 mM NaCl, 20 mM Imidazole, pH 7.4). Proteins were eluted in a stepwise manner using re-suspension buffer containing increasing concentrations of imidazole – 50 mM (5 mL), 100 mM (3 mL), 200 mM (3 mL) and 300 mM (3 mL). The presence of the protein of interest in fractions was confirmed by SDS-PAGE, and gel filtration (Superdex 75/200, GE Healthcare) was used for further purification using storage buffer (20 mM Tris-HCl, 100 mM NaCl pH 7.4) at a flow rate of 1 mL/min. Fractions containing the protein of interest were pooled and concentrated to 100 - 150 µM using a 30 kDa MWCO Viva-Spin centrifugal concentrator (Sartorius). Glycerol was added to the concentrated protein samples to a final concentration of 10 % (v/v) and they were snap-frozen in liquid N_2 and stored at -80˚C.

1.5 Minimum inhibitory concentration (MIC) measurements

Minimal inhibitory concentration measurements were carried out using the broth micro-dilution method, following CLSI guidelines[6]. Briefly, reference bacterial strains were grown overnight in Mueller-Hinton (MH) broth at 30 ºC. In a 96-well micro-titer plate, 50 µl of serial two-fold dilutions of gladiostatin in MH broth were mixed with 50 µl of bacterial suspension, diluted to a concentration of 106 CFU/ml in MH broth. The desired inoculum density was achieved using a 0.5 McFarland turbidity standard. Following incubation for 18h at 30 °C, MICs were defined as the lowest concentrations that visibly inhibited bacterial growth.

For the antifungal susceptibility testing against yeast strains, a reference method was used following the M27-A2 CLSI approved standard[7]. *S. cerevisiae* W303-1a was grown overnight in YPD at 30 ºC. The culture was resuspended in PBS and standardized using a 0.5 McFarland turbidity standard. The working solution was prepared using a 1:1000 dilution in RPMI (1 to 5 x 10^3 cells/mL). 0.1 ml of the final

culture dilution was added into each well. Drug dilutions were added to each well before the addition of the culture, using 0.1 µl of serial two-fold dilutions of gladiostatin in RPMI. Sterility control (no cells added) and growth control (DMSO control) were included in the 96-well plate. The plate was incubated independently without shaking at 35 °C and 37 °C for 24 h. All MIC determinations were performed in triplicate.

1.6 Cytotoxicity studies

The cancer cells were obtained from the European Collection of Cell Cultures and grown in Roswell Park Memorial Institute medium (RPMI 1640) supplemented with 10 of foetal calf serum, 1 of 2 mM glutamine and 1 penicillin/streptomycin using a 5 % CO_2 humidified atmosphere and passaged at approximately 70 - 80% confluence. Cells were seeded in a 96 well plate at a density of 5000 cells per well. After 48 h of pre-incubation time in drug free medium at 37 ºC, various concentrations of gladiostatin were added. A stock solution of the compound was firstly prepared in 5% DMSO (v/v) and 95 % cell culture medium (v/v) following serial dilutions in RPMI 1640. After 24 h of exposure, supernatants were removed and wells were washed with PBS before adding fresh medium. Following 72 h of recovery time in drug free medium and 37 ºC, cell viability was assessed using the MTT assay. Formazan absorbance at 570 nm was recorded in a FLUOstar Omega microplate reader. Values were determined as duplicates of triplicates in two independent sets of experiments and their standard deviations were calculated. For SKOV-3 ovarian, HCT166 p53-/- colorectal and MIA Paca2 pancreatic cancer cell lines, the same protocol was followed, except that the cells were grown in McCoy's 5A modified medium (SKOV-3 and HCT165 p%3-/-) and Dulbecco's modified Eagle medium (high glucose; MIA Paca2).

1.7 Wound healing assay

24 well plates were inoculated with A2780 ovarian cancer cells (10,000 cells/well) and allowed to reach 90% confluence. Following attachment, two "wounds" were created in each well using a pipette tip and cells were treated with 240 nM gladiostatin (**6**). After 24 h of exposure, the solution of gladiostatin (**6**), was removed by suction, cells were washed with PBS and stained using crystal violet solution prepared with 10 % ethanol. Excess stain was washed with PBS and cells were visualised using a 4x transmission microscope.

1.8 Cladogram construction

The ACP cladogram was generated in a similar manner to previous reports[8]. Well-characterised *trans*-AT PKSs were extracted from the MiBiG repository: (albicidin, Alb, BGC0001088; bacillaene, Bae, BGC0001089; batumin, Bat, BGC0001099; bongkrekic acid, Bon, BGC0000173; bryostatin, Bry, BGC0000174; calyculin, Cal, BGC0000967; chivosazole, Chi, BGC0001069; corallopyronin, Cor, BGC0001091; cycloheximide, Chx, BGC0000175; difficidin, Dif, BGC0000176; disorazole, Dsz, BGC0001093; elansolid, Ela, BGC0000178; enacyloxin, Ena, BGC0001094; etnangien, Etn, BGC0000179; kirromycin, Kir, BGC0001070; lactimidomycin, Ltm, BGC0000083; leinamycin, Lnm, BGC0001101; macrolactin, Mln, BGC0000181; migrastatin, Mgs, BGC0000177; nosperin, Nsp, BGC0001071; oocydin, Ooc, BGC0001031; onnamide, Onn, BGC0001105; psymberin, Psy, BGC0001110; rhizopodins, Riz, BGC0001111; rhizoxins, Rhi, BGC0001112; sorangicin, Sor, BGC0000184; thailandamide, Tai, BGC0000186, and thailanstatin, Tst, BGC0001114)[9]. ACP cladograms were rooted using representative domains from *cis*-AT assembly lines (amphotericin module 10, Amp10, BGC0000015; concanamycin module 6, Con6, BGC0000040; erythromycin module 4, Ery4, BGC0000055; mycolactone module 4, Myc4, BGC0000103; nystatin module 9, Nys9, BGC0000115; rapamycin module 6, Rap6, BGC0001040; rifamycin module 5, Rif5, BGC0000136; spinosad module 5, Spn5, BGC0000148; and tylosin module 3, Tyl3, BGC0000166)[9]. Sequences were aligned using the ClustalX multiple sequence alignment software and RaxML- NG for maximum likelihood phylogenetic inference [10,11]. The cladogram was visualised using FigTree v1.4.4 software.

1.9 Butenolide formation assay

The buffer used for storage of the His_8-ACP-PBS protein was used for the enzymatic reactions. 400 µM of 3-ketooctanoyl NAC thioester, 400 µM of DHAP and 124.3 µg of the His_8-ACP-PBS protein in a total volume of 200 µL were incubated for 90 min at room temperature. One unit of shrimp alkaline phosphatase (New England Biolabs) was added and the resulting mixture was incubated for 45 min i at 30˚C. 200 µL of methanol was added and the mixture was allowed to stand for 5 min. After centrifugation at 14, 000 rpm for 10 min, the supernatant was filtered (0.4 µm) prior to UHPLC-ESI-Q-TOF-MS analysis.

1.10 Synthesis of butenolide standard and 3-keto-octanoyl-SNAc

The butenolide standard and 3-keto-octanoyl-SNAc were synthesised according to established protocols[12,13].

1.11 Assay for chain release from the ACP-PBS didomain

The ACP domain of the *apo*-ACP-PBS didomain (100 µM) was loaded with the phosphopantetheine thioester of acetoacetate by incubation with 2 µM Sfp, 1 mM acetoacetyl-CoA (Sigma-Aldrich) and 10 mM $MgCl_2$ in 20 mM Tris, 100 mM NaCl (total volume 50 µL) for 1 hr at RT. Excess acetoacetyl-CoA was removed by two rounds of 10-fold dilution with 20 mM Tris, 100 mM NaCl and concentration to 50 µL using a 10 kDa MWCO VivaSpin centrifugal concentrator. DHAP (400 µM) was added and the mixture was left to react for 90 min at room temperature. The reaction (and a control from which DHAP was omitted) were diluted 5-fold with water and analysed by UHPLC-ESI-Q-TOF-MS.

1.12 UHPLC-ESI-Q-TOF-MS analysis of intact pHis_8-ACP-PBS

All intact protein mass spectrometry analyses were conducted on a Bruker MaXis II ESI-Q-TOF-MS connected to a Dionex 3000 RS UHPLC fitted with an ACE C4-300 RP column (100 x 2.1 mm, 5 µm, 30 °C). The column was eluted with a linear gradient of 5–100% MeCN containing 0.1% formic acid over 30 min. The mass spectrometer was operated in positive ion mode with a scan range of 200–3000 *m/z*. Source conditions were: end plate offset at −500 V; capillary at −4500 V; nebulizer gas (N_2) at 1.8 bar; dry gas (N_2) at 9.0 L min^{-1}; dry temperature at 200 °C. Ion transfer conditions were: ion funnel RF at 400 Vpp; multiple RF at 200 Vpp; quadrupole low mass at 200 *m/z*; collision energy at 8.0 eV; collision RF at 2000 Vpp; transfer time at 110.0 µs; pre-pulse storage time at 10.0 µs.

2. Supplementary Figures

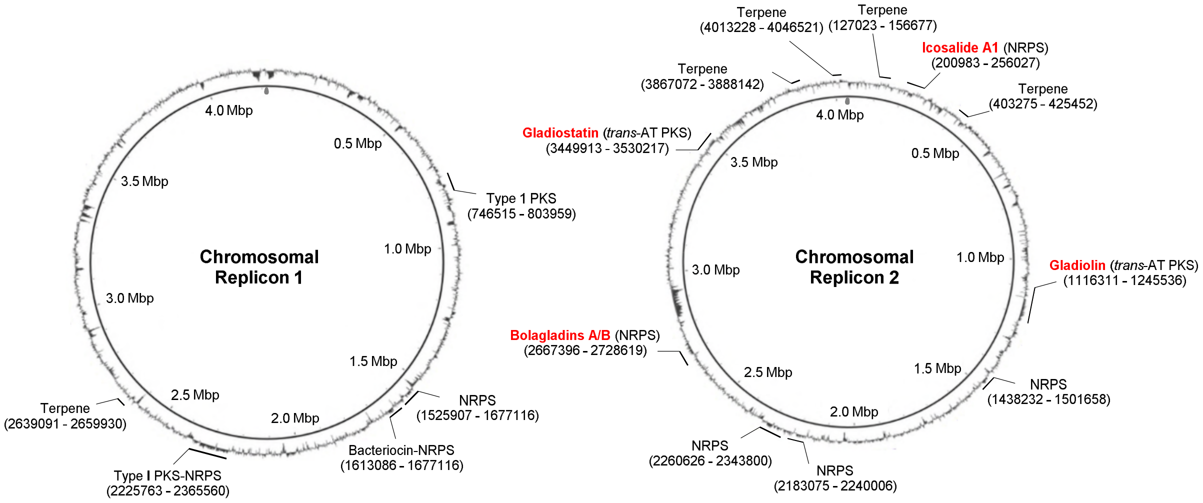

Figure S1. Genome structure of *B. gladioli* BCC0238 highlighting the location of specialised metabolite biosynthetic gene clusters. The two large circular chromosomal replicons of *B. gladioli* BCC0238 are shown. Base pairs are numbered inside each circle and the relative GC-content is indicated around the outside. BGCs encoding various types of specialised metabolite biosynthetic system are indicated and the base pair range each occupies is shown in parentheses. In cases where the product of the BGC has been identified, the name of the metabolite is given (in red). The gladiostatin (*gds*) BGC reported in this study is located on the second chromosome at approximately 3.5 Mbp.

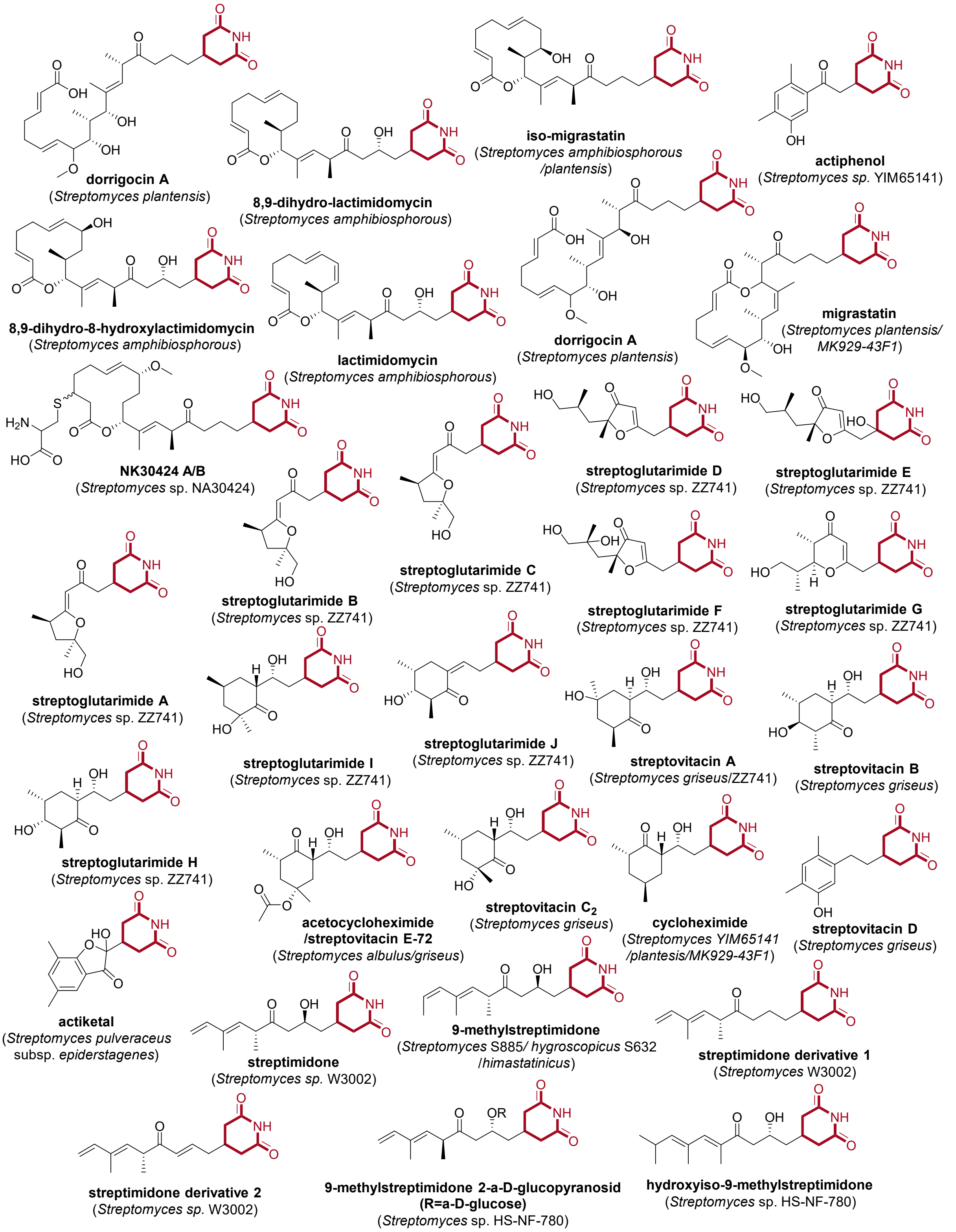

Figure S2. Structures of known glutarimide antibiotics. The common 2, 6-piperidinedione moiety presented in all metabolites is highlighted in red[14–25]. The organisms they were isolated from are given below the compound names (all *Streptomyces* species)**.**

a

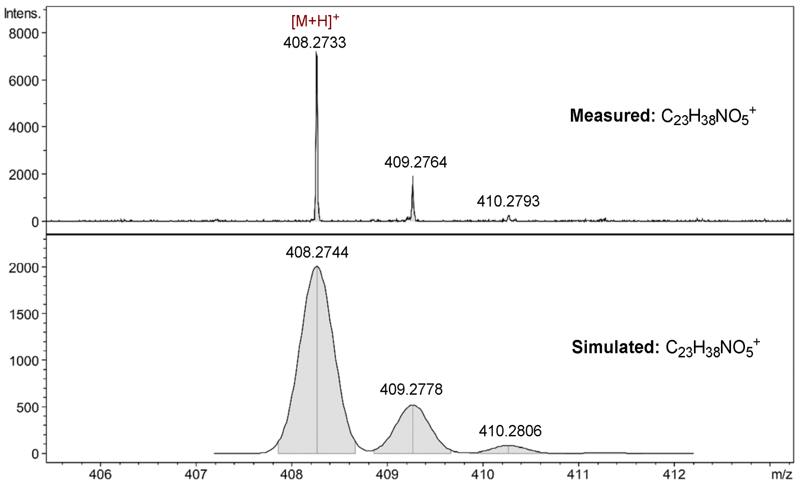

b

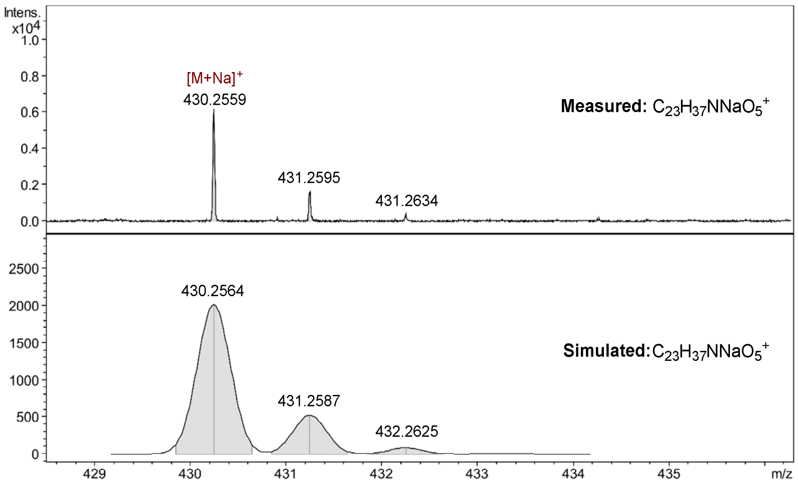

Figure S3. High resolution mass spectra the of gladiostatin degradation product (5). **a**) Measured (top) and simulated (bottom) spectra for the $[M+H]^+$ ion. **b**) Measured (top) and simulated (bottom) spectra for the $[M+Na]^+$.

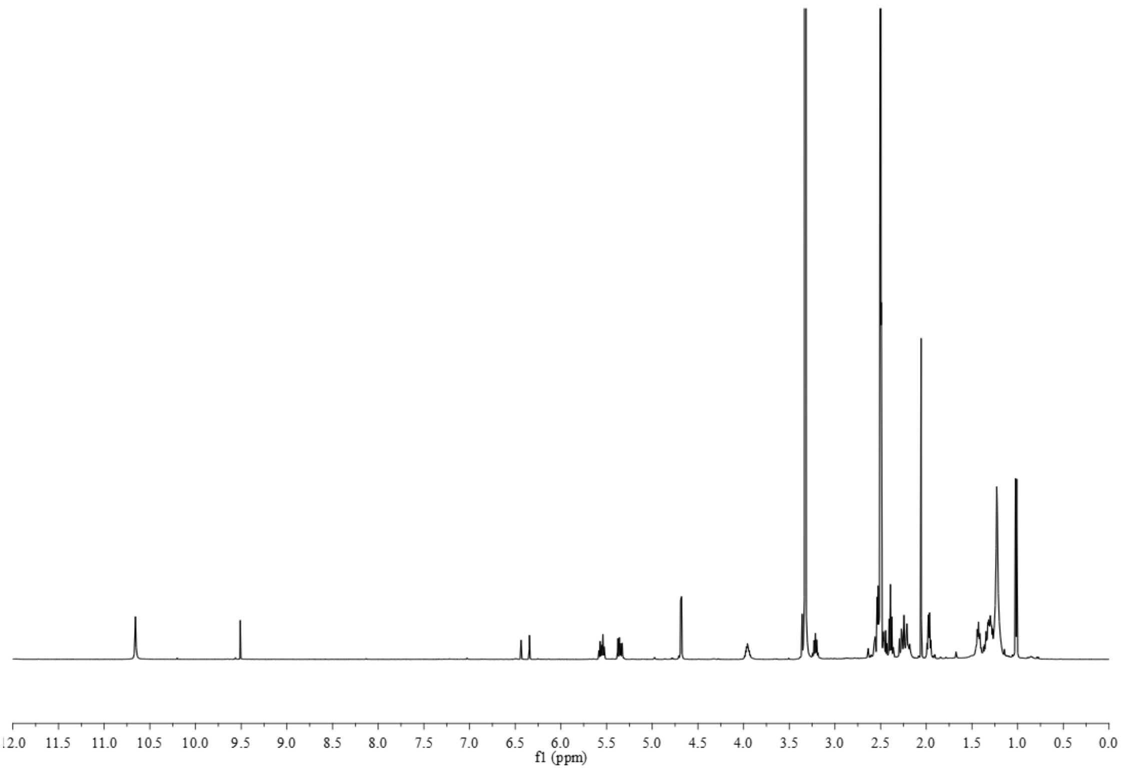

Figure S4. ^{1}H NMR spectrum of degradation product (5) in DMSO-d_6.

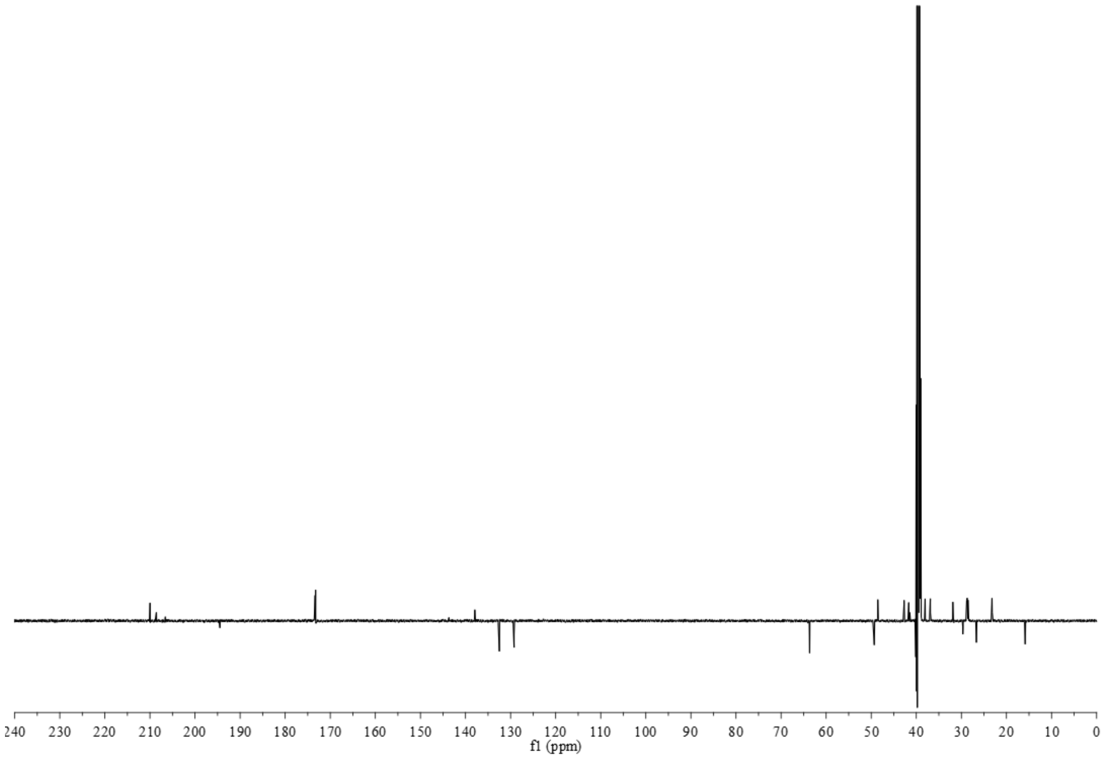

Figure S5. ^{13}C NMR spectrum of degradation product (5) in DMSO-d_6.

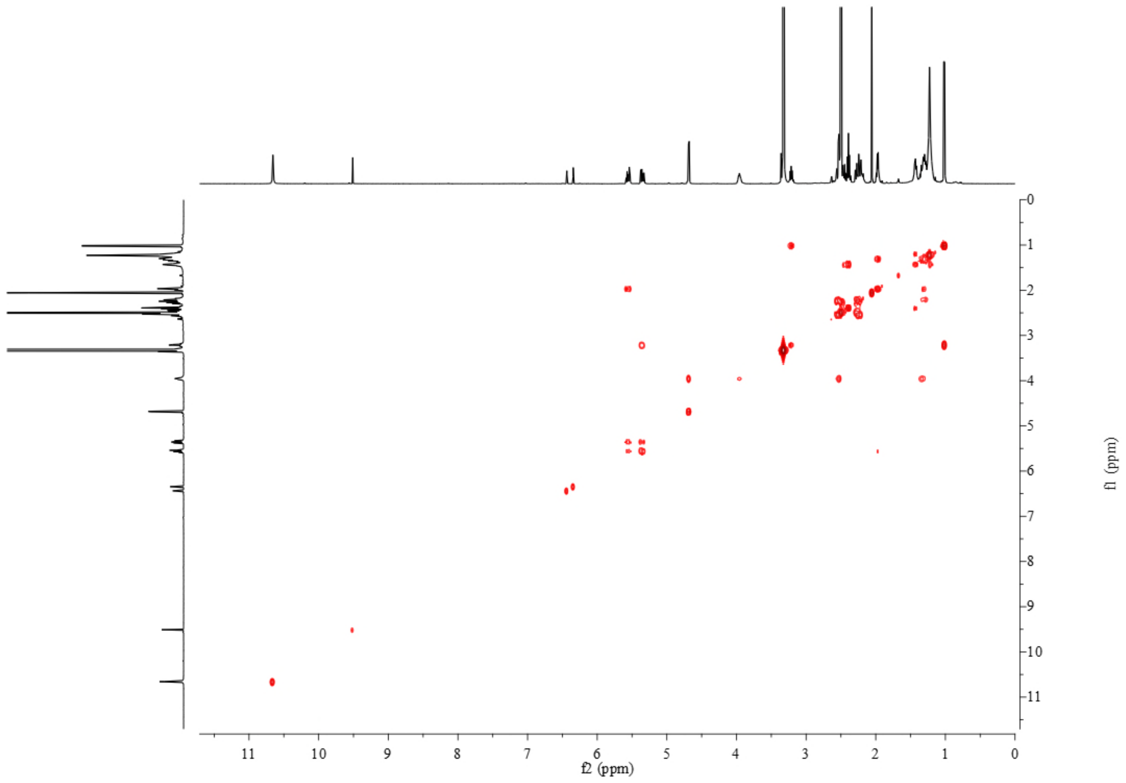

Figure S6. COSY NMR spectrum of degradation product (5) in DMSO-d_6.

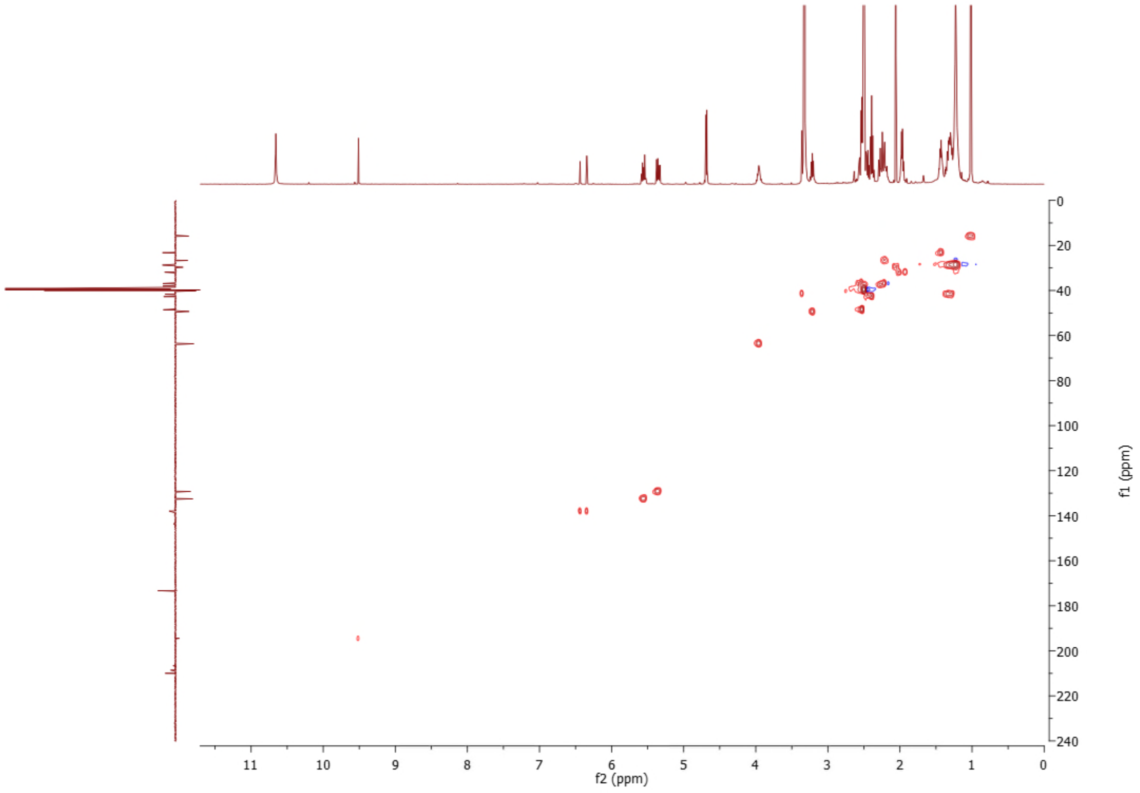

Figure S7. HSQC NMR spectrum of degradation product (5) in DMSO-d_6.

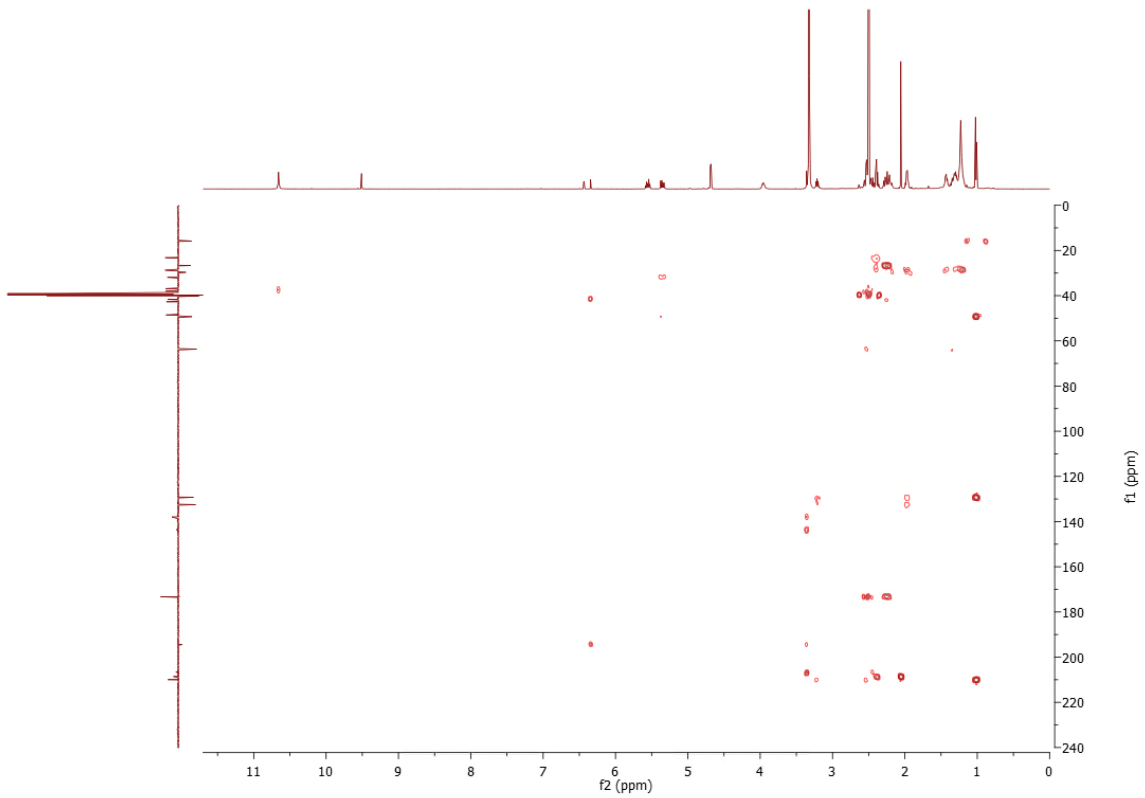

Figure S8. HMBC NMR spectrum of degradation product (5) in DMSO-d$_6$.

a

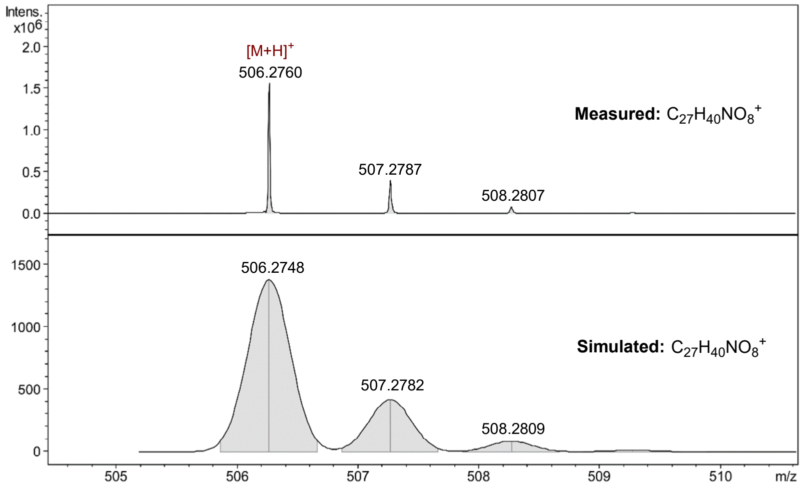

b

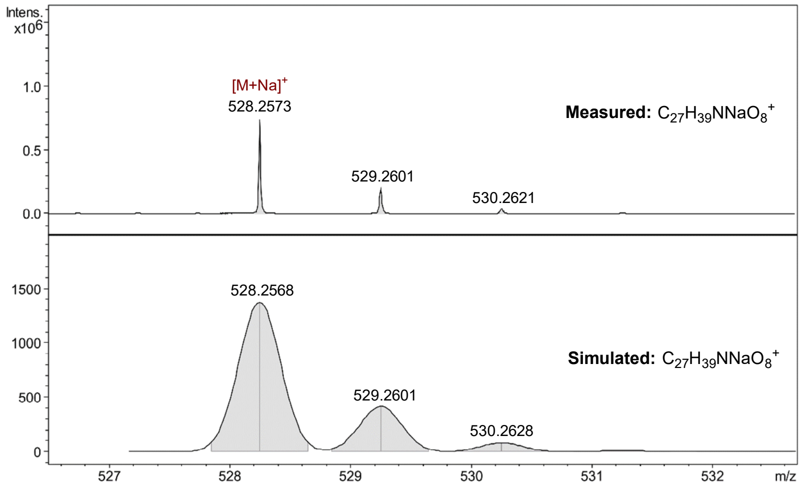

Figure S9. High resolution mass spectra of gladiostatin (6). **a**) Measured (top) and simulated (bottom) spectra for the $[M+H]^+$ ion. **b**) Measured (top) and simulated (bottom) spectra for the $[M+Na]^+$ ion.

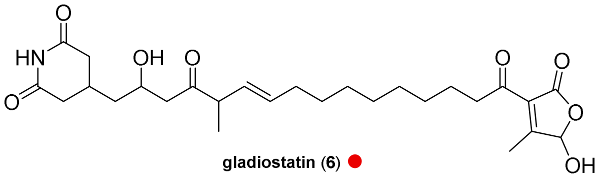

gladiostatin (6)

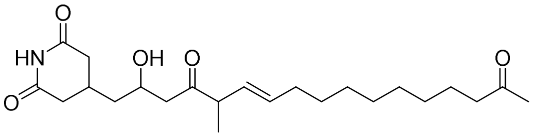

degradation product (5)

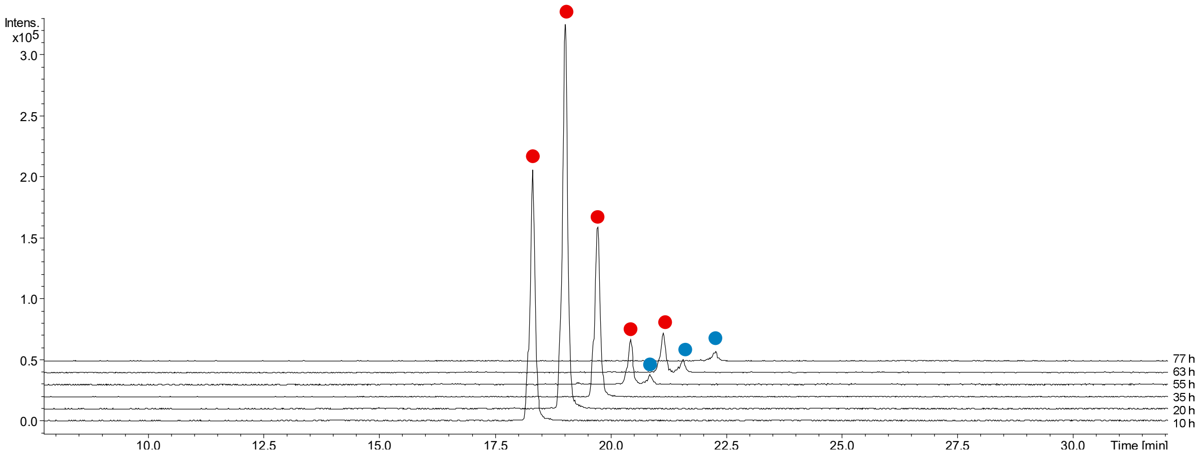

Figure S10. Time-course analysis of gladiostatin production. Extracted ion chromatograms at *m/z* = 506.27 ± 0.02 (corresponding to gladiostatin – red circles) and 408.27 ± 0.02 (corresponding to degradation product **5** – blue circles) from UHPLC-ESI-Q-TOF-MS analyses of ethyl acetate extracts of *B. gladioli* BCC1622 grown for different lengths of time (indicated at the right of each chromatogram). At time points beyond 35 hours, other low intensity ions can be observed with molecular formulae that are related to gladiostatin. These likely correspond to additional degradation products.

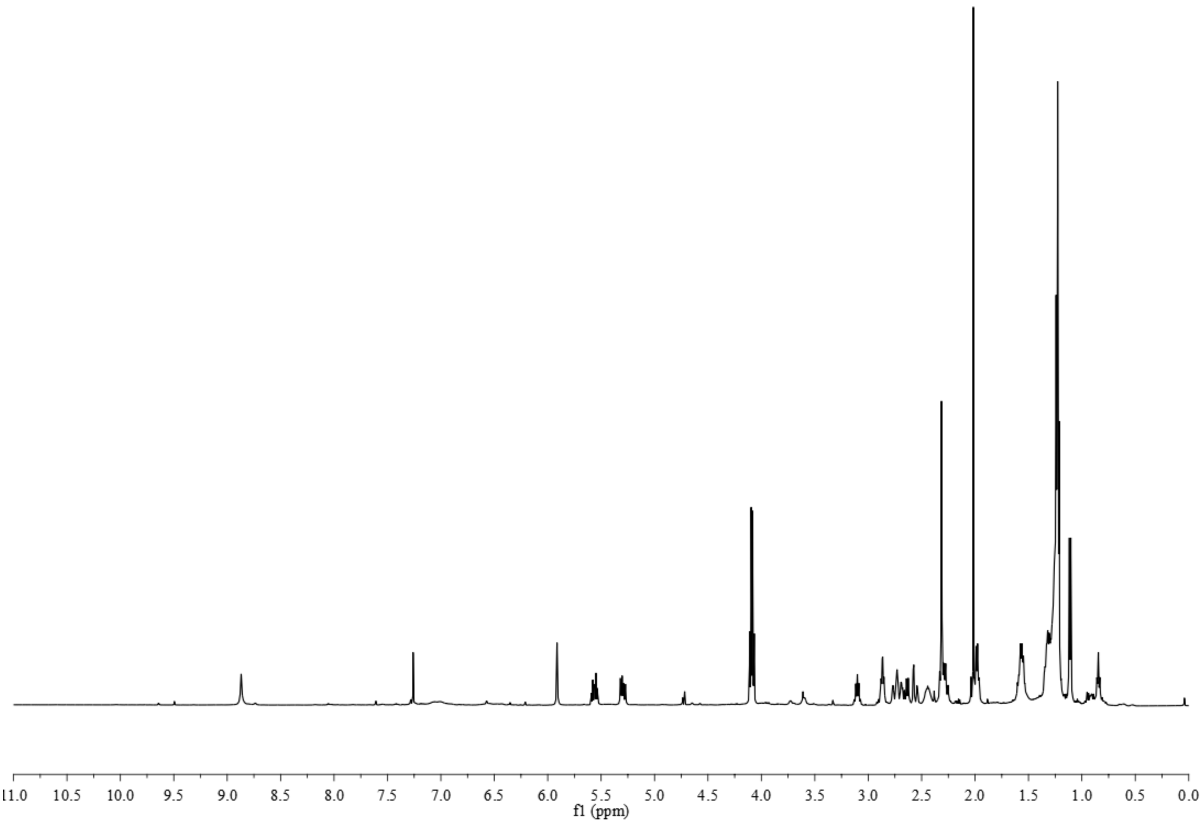

Figure S11. ^{1}H NMR spectrum of gladiostatin (6) in $CDCl_3$.

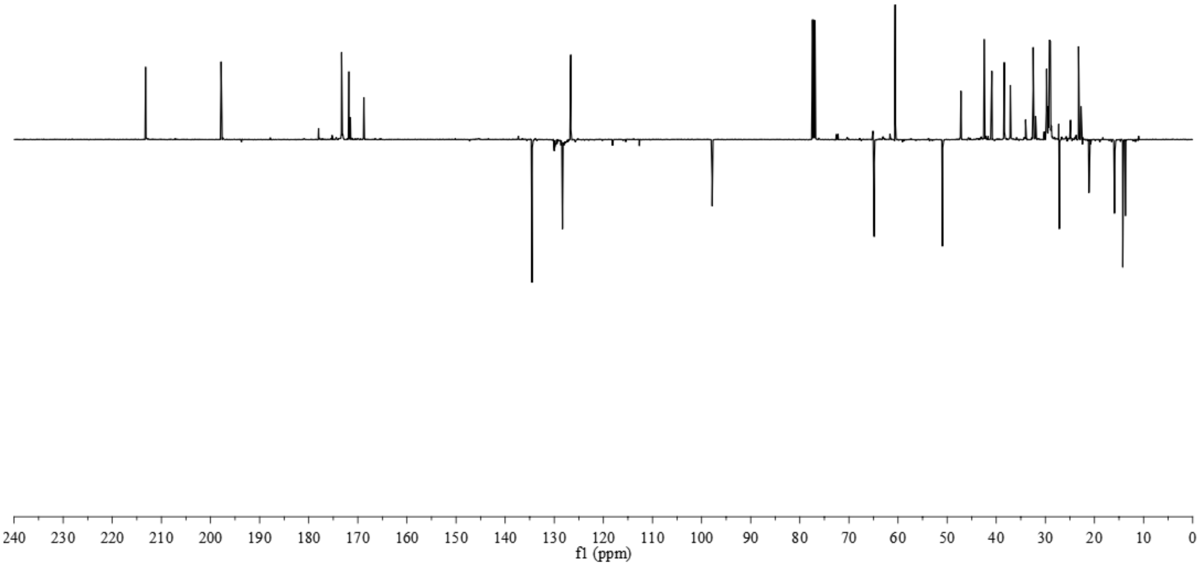

Figure S12. ^{13}C NMR spectrum of gladiostatin (6) in $CDCl_3$.

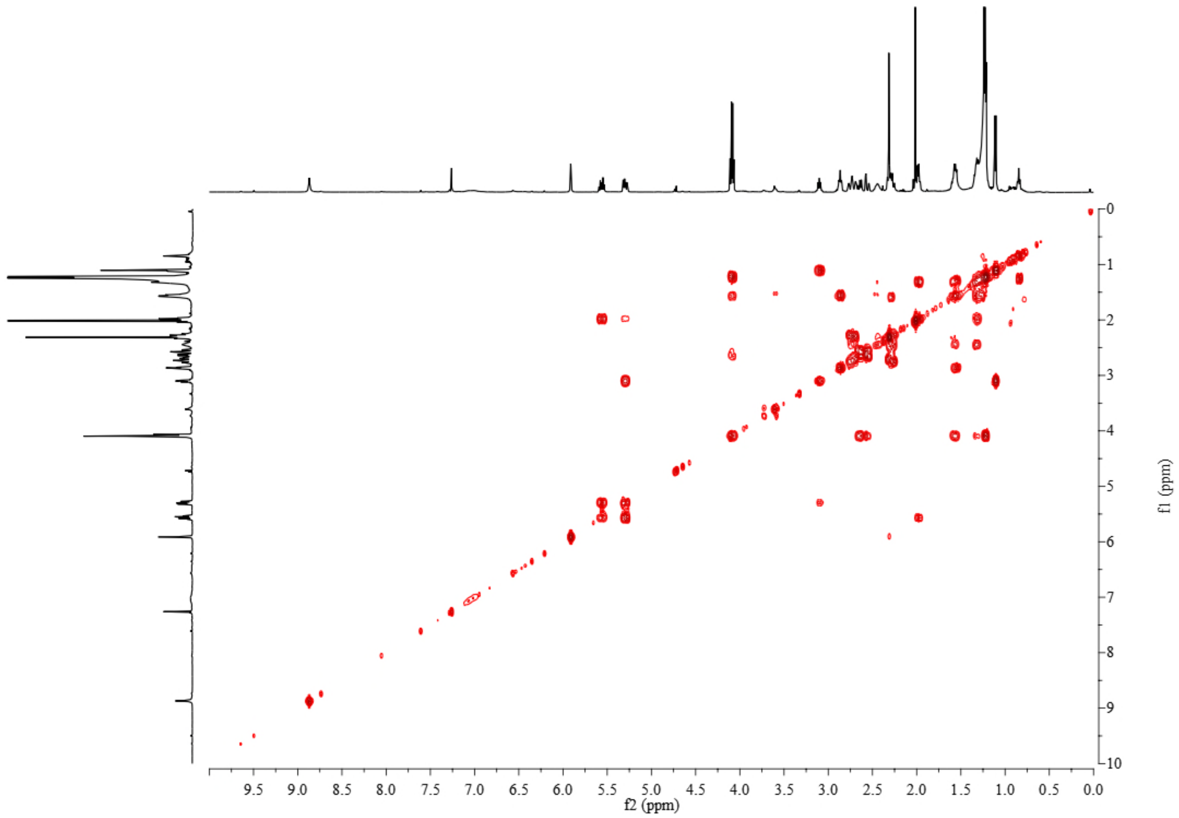

Figure S13. COSY NMR spectrum of gladiostatin (6) in $CDCl_3$.

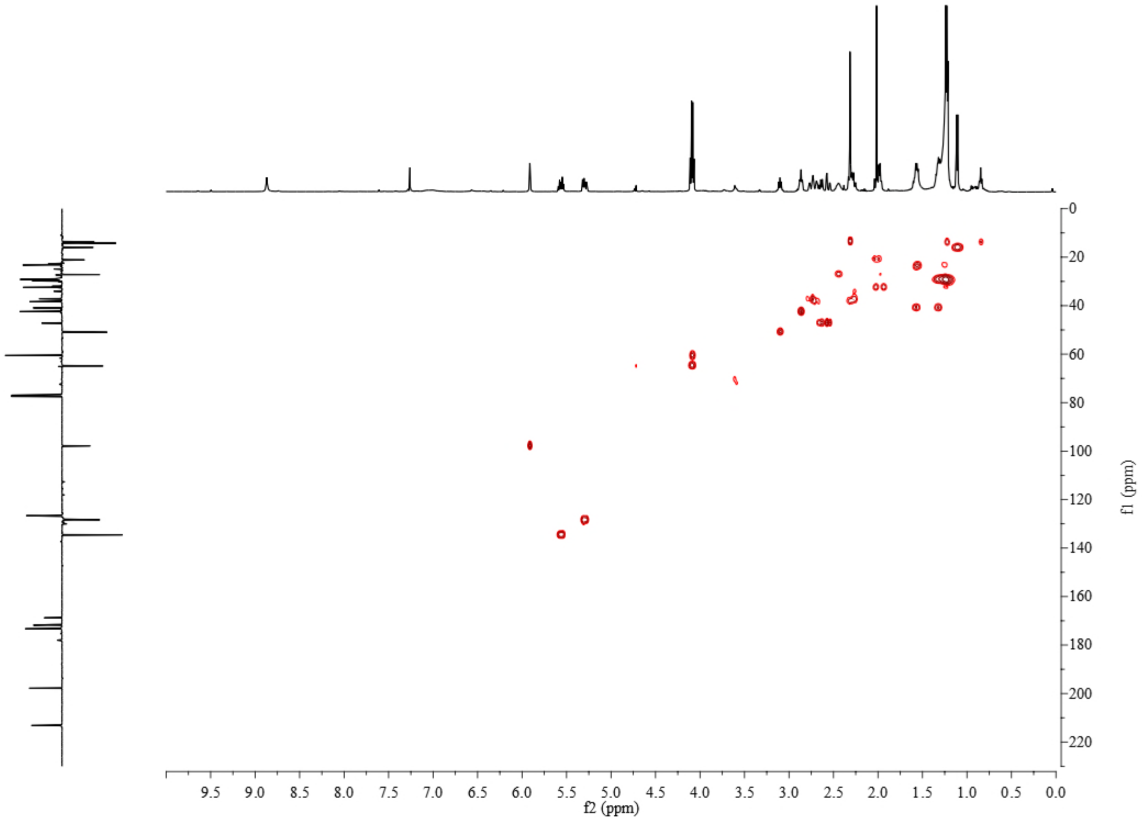

Figure S14. HSQC NMR spectrum of gladiostatin (6) in $CDCl_3$.

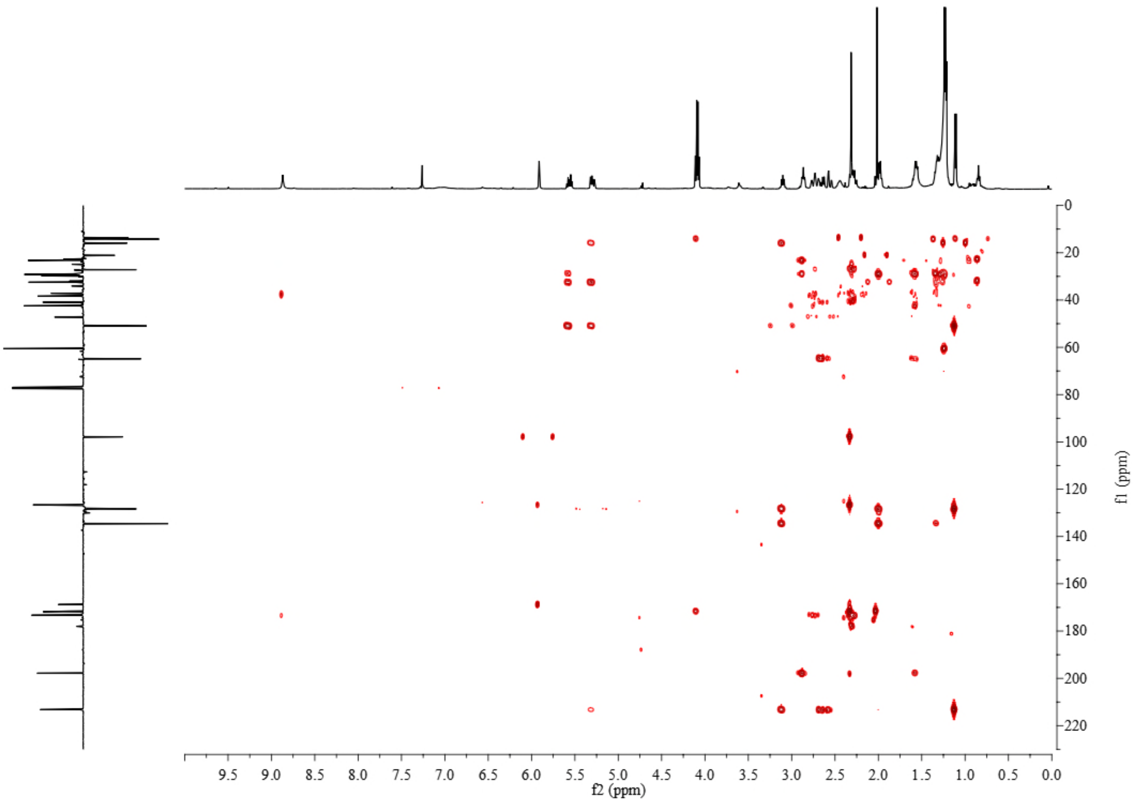

Figure S15. HMBC NMR spectrum of gladiostatin (6) in $CDCl_3$.

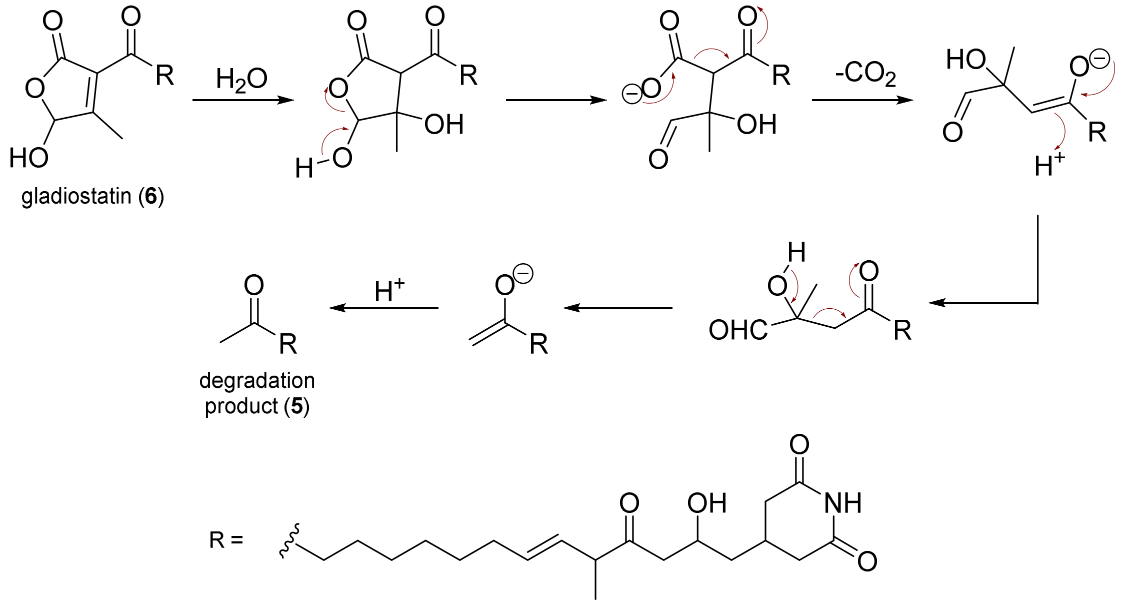

Figure S16. Proposed mechanism for conversion of gladiostatin (6) to degradation product (5).

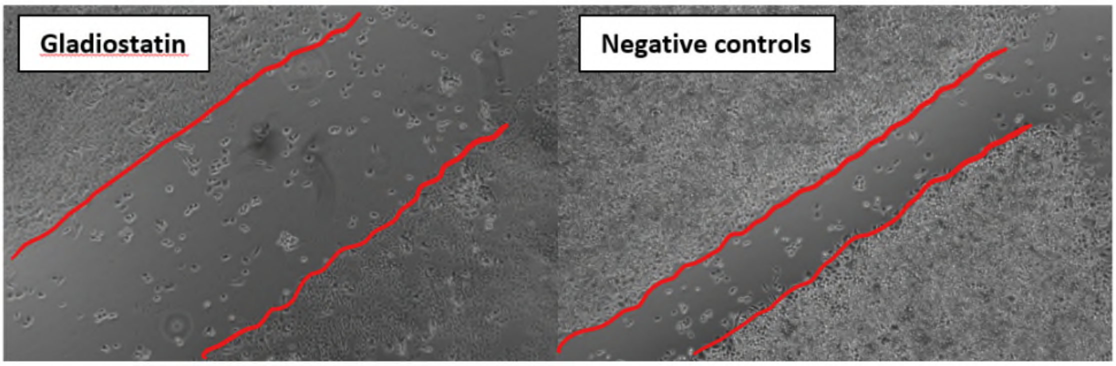

Figure S17. Inhibition of A2780 ovarian tumour cell migration by gladiostatin. Microscope images showing wound-width after 24 hours of wound induction for untreated negative control cells and cells treated with 240 nM gladiostatin (**6**).

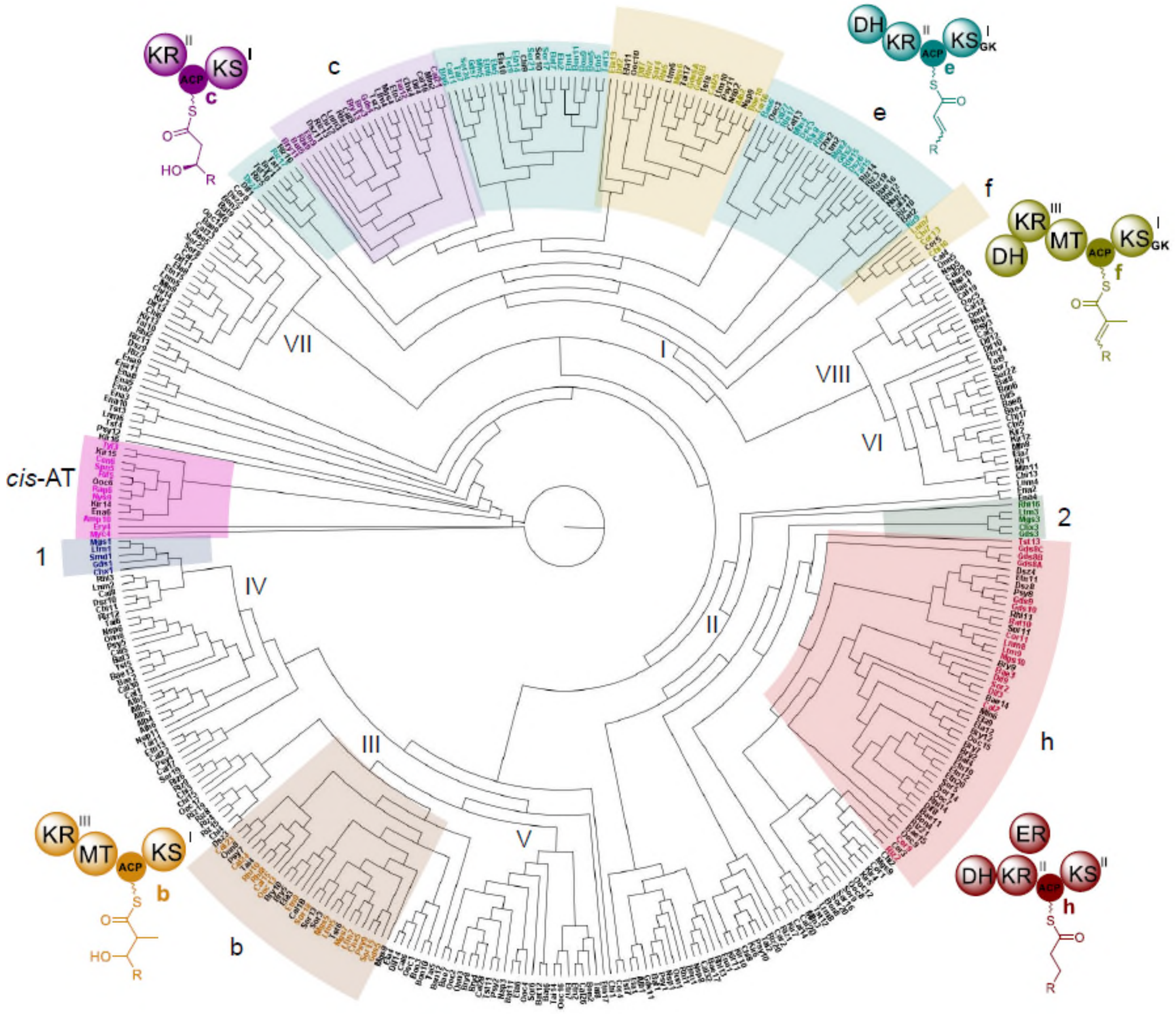

Figure S18. Cladogram comparing the sequences of ACP domains from various *trans*-AT PKSs with the ACP domains of the gladiostatin PKS. Domain architectures for the module types observed in the gladiostatin PKS are highlighted and illustrated. A selection of ACP domains from *cis*-AT PKSs were used to root the tree and clade notation is as described by Vanderwood *et al.*[8] In addition to the ACP domain sequences previously analysed Vanderwood *et al.*, sequences of ACP domains from branching sub-modules (KS-B-ACP) and stand-alone ACPs involved in starter unit biosynthesis were included. This resulted in to two new clades (1 and 2).

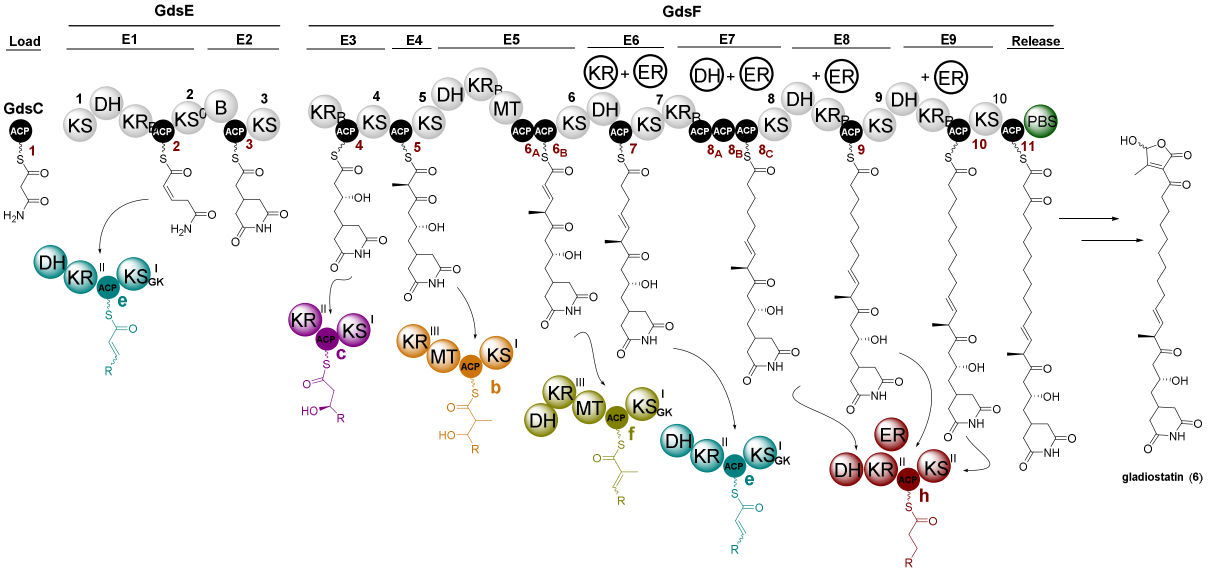

Figure S19. Analysis of evolutionary units comprising the gladiostatin PKS. Evolutionary units (E) and ACPs are numbered (1-9 and 1-11, respectively). Arrows indicate the predicted phylogenetic classifiers. Gate keeping (GK) catalytic domains are highlighted.

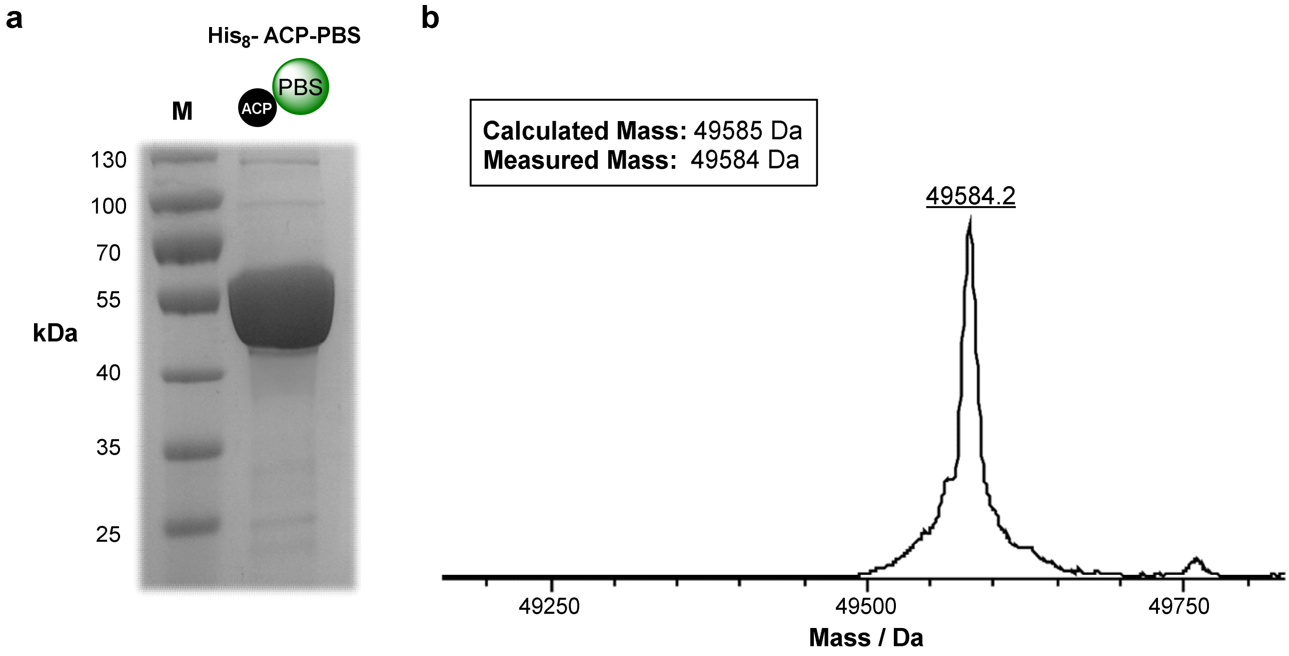

Figure S20. Analysis of recombinant His$_8$-ACP-PBS di-domain by SDS-PAGE and MS. **a**) 10% SDS-PAGE gel containing purified His$_8$-ACP-PBS di-domain. **b**) Deconvoluted mass spectrum of intact His$_8$-ACP-PBS di-domain. Calculated and measured masses are displayed.

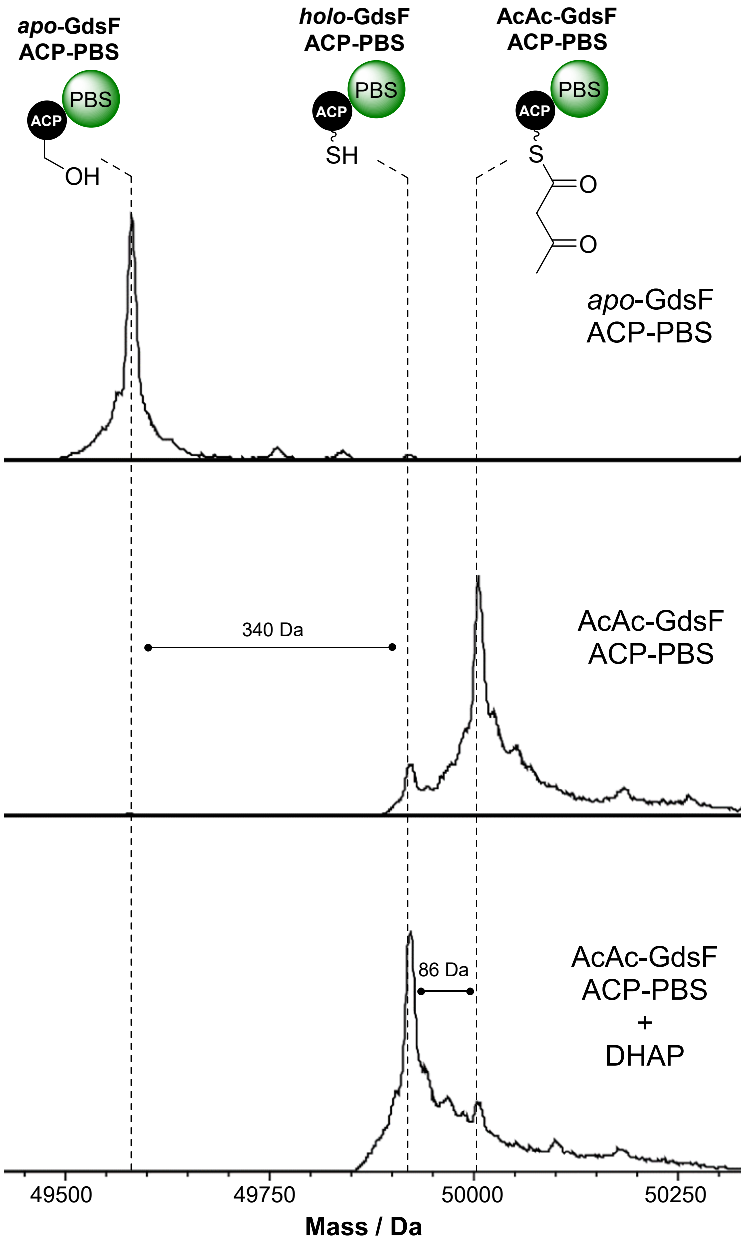

Figure S21. Mass spectrometric analysis of acetoacetyl thioester release from the ACP-PBS di-domain by DHAP. Stacked deconvoluted mass spectra of: *apo*-His_8-ACP-PBS di-domain (top), PPTase-catalysed loading of acetoacetyl-phosphopantetheine onto the ACP domain from acetoacetyl-CoA (middle), and release of the acetoacetyl unit upon incubation with DHAP, yielding the *holo*-ACP-PBS di-domain (bottom).

3. Supplementary Tables

Table S1. ^{1}H and ^{13}C NMR data for degradation product (**5**) in DMSO-d_6.

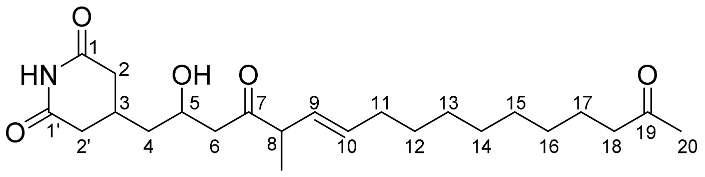

degradation product (5)

Position	δ_C (ppm)	δ_H (ppm)	HMBC
1	173.2	-	-
1-NH	-	10.66 (s)	C-1, C-2, C-2’
2	36.9	2.24 (m)/2.25(m)	C-1, C-3, C-2’, C-4
3	26.67	2.20	C-1, C-2, C-2’, C-1’, C-4
2’	38.0	2.27 (m) /2.50 (m)	C-3, C-2, C-1’, C-4
1’	173.3	-	-
4	41.67	1.35 (m)	C-2, C-3, C-2’, C-5, C-6
5	63.65	3.96 (m)	C-3, C-4, C-6, C-7
6	48.54	2.52 (m)	C-4, C-5, C-7, C-8
7	209.97	-	-
8	49.32	3.21 (m)	C-7, C-9, C-10, C-8Me
8-Me	15.8	1.07 (d, J = 6.8 Hz)	C-7, C-8, C-9
9	129.23	5.35 (dd, J = 15.3, 8.2)	C-8, C-10, C-11, C-8Me
10	132.49	5.56 (m)	C-8, C-9, C-11
11	31.9	1.97 (q, J = 6.8 Hz)	C-9, C-10
12	28.44-28.83	1.23	-
13	28.44-28.83	1.23	-
14	28.44-28.83	1.23	-
15	28.44-28.83	1.23	-
16	28.44-28.83	1.23	-
17	23.2	1.43 (m)	C-18, C-19
18	42.7	2.39 (t, J = 7.4 Hz)	C-17, C-19, C-20
19	208.5	-	-
20	29.68	2.06 (s)	C-19

Table S2. ^{1}H and ^{13}C NMR data for gladiostatin (**6**) in $CDCl_3$.

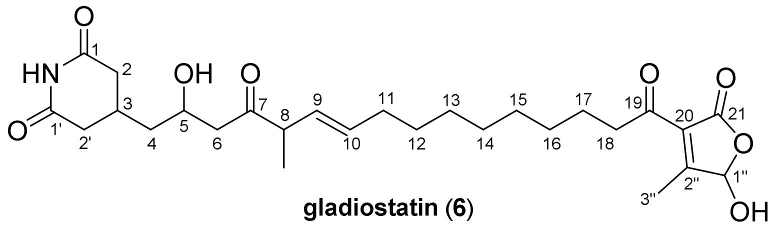

gladiostatin (6)

Position	δ_C (ppm)	δ_H (ppm)	HMBC
1	173.3	-	-
1-NH	-	8.87 (s)	C-1, C-1', C-2, C-2'
2	37.73	2.27 (m) / 2.73 (m)	C-1, C-3, C-2', C-4
3	27.15	2.44 (m)	C-1, C-2, C-2', C-1', C-4
2'	38.37	2.32 (m) / 2.71 (m)	C-3, C-2, C-1', C-4
1'	173.2	-	-
4	40.90	1.33 (m) / 1.57 (m)	C-2, C-3, C-2', C-5, C-6
5	64.86	4.09 (q, *J* = 7.2 Hz)	C-3, C-4, C-6, C-7
6	47.22	2.57 (m)	C-4, C-5, C-7, C-8
7	213.21	-	-
8	50.96	3.09 (d, *J* = 7.4 Hz)	C-7, C-9, C-10, C-8Me
8-Me	15.93	1.11 (d, *J* = 6.8 Hz)	C-7, C-8, C-9
9	128.33	5.29 (m)	C-8, C-10, C-11, C-8Me
10	134.56	5.56 (dt, *J* = 15.4, 6.8 Hz)	C-8, C-9, C-11
11	32.47	1.98 (t, *J* = 7.2 Hz)	C-9, C-10
12	28.67-29.85	1.23	-
13	28.67-29.85	1.23	-
14	28.67-29.85	1.23	-
15	28.67-29.85	1.23	-
16	28.67-29.85	1.23	-
17	23.24	1.56 (m)	C-18, C-19
18	42.46	2.86 (t, *J* = 7.2 Hz)	C-17, C-19, C-20
19	197.84	-	-
20	126.63	-	-
21	171.82	-	-
1''	97.84	5.91 (s)	C-20, C-2'', C-3''
2''	168.77	-	-
3''	13.69	2.31(s)	C-20, C-2'', C-1''

Table S3. Putative functions of proteins encoded by genes in the gladiostatin biosynthetic gene cluster.

Gene/Protein	Length bp/aa	Similar Proteins	% Identity
gdsA/GdsA	699/233	Phosphoglycerate mutase (*Collimonas arenae*)<br>Histidine phosphatase protein family (*Myxococcus xanthus*)<br>Histidine phosphatase family protein (*Sinobacteraceae bacterium*)	50<br>44<br>42
gdsB/GdsB	3339/1113	ACP S-malonyltransferase (*Bacillus cereus*)<br>ACP S-malonyltransferase (*Bacillus subtilis*)<br>Malonyl CoA-ACP transacylase (*Paenibacillus riograndensis*)	50<br>48<br>47
gdsC/GdsC	240/80	Acyl carrier protein (*Streptomyces eurocidicus*)<br>Acyl carrier protein (*Streptomyces* sp. W007)<br>Acyl carrier protein (*Pseudomonas syringae*)	55<br>52<br>42
gdsD/GdsD	1995/665	Asparagine synthase (glutamine-hydrolyzing) (*Robbsia andropogonis*)<br>Asparagine synthase (glutamine-hydrolyzing) (*Streptomyces griseus*)<br>Asparagine synthase (glutamine-hydrolyzing) (*Stappia indica*)	79<br>61<br>58
gdsE/GdsE	9744/3248	SDR family NAD(P)-dependent oxidoreductase (*Robbsia andropogonis*)<br>Polyketide synthase (Streptomyces olivoreticuli)<br>ChxE (*Streptomyces* sp. YIM 56141)	67<br>49<br>47
gdsF/GdsF	30543/10181	Polyketide synthase PksN (*Methylomusa anaerophila*)<br>Non-ribosomal peptide synthase (*Bacillus swezeyi*)<br>Polyketide synthase PksN (*Paenibacillus halotolerans*)	49<br>41<br>40
gdsG/GdsG	711/237	HAD family phosphatase (*Streptomyces* sp. BK329)<br>Phosphoglycolate phosphatase-like HAD superfamily hydrolase (*Streptomyces rochei*)<br>HAD family hydrolase (*Streptococcus thermophilus*)	44<br>44<br>43
gdsH/GdsH	1020/340	NADP-dependent oxidoreductase (*Paucibacter toxinivorans*)<br>NADP-dependent oxidoreductase (*Pelomonas saccharophila*)<br>NADP-dependent oxidoreductase (*Xylophilus ampelinus*)	65<br>65<br>60
gdsI/GdsI	906/302	LysR family transcriptional regulator (*Caballeronia calidae*)<br>LysR family transcriptional regulator (*Caballeronia sordidicola*)<br>LysR family transcriptional regulator (*Paraburkholderia caribensis*)	80<br>74<br>72

Table S4. Substrate specificities of KS domains in the gladiostatin PKS predicted by the TransATor webserver[26].

KS domain	Substrate specificity
GdsEKS1	unusual starter (AMT/succinate)
GdsEKS2	non-elongating (double bonds (mostly *Z*-configured)
GdsEKS3	vinylogous chain branching
GdsFKS4	*β*-D-OH (some with α-Me)
GdsFKS5	α-Me reduced/keto/D-OH
GdsFKS6	α-Me double bonds
GdsFKS7	double bonds (mostly *E*-configured)
GdsFKS8	completely reduced
GdsFKS9	completely reduced
GdsFKS10	completely reduced

Table S5. Predicted stereospecificities of KR domains in the gladiostatin PKS. Sequence alignments and comparisons with KR domains from *trans*-AT PKSs with known stereospecificities were used to make the predictions.

KR domain	Diagnostic Asp Motif	KR type	Resulting stereochemistry
KR1	AGVL--Q**D**GLI	B type (*D*)	-
KR2	AGTL--R**D**GFA	B type (*D*)	(*R*)
KR3	AIVL--Q**D**RTL	B type (*D*)	-
KR4	AGFL--R**D**AYL	B type *(D)*	-
KR5	AGLL--R**D**AYL	B type *(D)*	-
KR6	AGLL--R**D**AYL	B type *(D)*	-

Table S6. Comparison of IC_{50} values for gladiostatin, lactimidomycin, migrastatin and cycloheximide against various cancer cell lines.

Human cancer cell line	gladiostatin	lactimidomycin	migrastatin	cycloheximide
lung	no activity [A549]	-	95 μM [Ms-1][27]	-
breast	-	0.004-0.05 μM [MDA-MB-231][21,28,29]	-	0.3 μM [MDA-MB-231][30], [MCF7][30]
ovarian	0.24 μM [A2780], 0.6 μM [SKOV3], 1.4 μM [PEA1]	-	-	-
esophageal	-	-	167 μM [EC17][27], >200 μM [EC109][27], >200 μM [HCE7][27], 138 μM [TT][27]	-
colon/colorectal	0.82 μM [HCTT116 P53 -/-]	0.102 μM [Moser][31], 0.018 μM [LoVo][28], 0.003 μM [HCT-116][31]	85 μM [LoVo][27], >200 μM [HT-29][27], 61 μM [SW480][27]	0.82 μM [HCT-15][30]
epidermoid	-		132 μM [A431][27]	-
epithelial	-	<0.0001 μM [HMEC][29]	-	-
murine melanoma	-	0.065 μM [B16-F10][31]	-	-
prostate	-	-	-	1.86 μM [Du145][30]
pancreatic	0.57 μM [MiaPaca2]	-	-	-
liver	-	-	-	0.57 μM [HepG2][32]

Table S7. Evolutionary classification of ACP domains in the gladiostatin PKS.[8]

ACP Domains	Module Type
Gds_ACP$_{1}$	undefined module type (***z***)
Gds_ACP$_{2}$	Family I (***e***)
Gds_ACP$_{3}$	undefined module type (***z***)
Gds_ACP$_{4}$	Family I (***c***)
Gds_ACP$_{5}$	Family III (***b***)
Gds_ACP$_{6A}$	Family I (***f***)
Gds_ACP$_{6B}$	Family I (***f***)
Gds_ACP$_{7}$	Family I (***e***)
Gds_ACP$_{8A}$	Family II (***h***)
Gds_ACP$_{8B}$	Family II (***h***)
Gds_ACP$_{8C}$	Family II (***h***)
Gds_ACP$_{9}$	Family II (***h***)
Gds_ACP$_{10}$	Family II (***h***)
Gds_ACP$_{11}$	undefined module type (***z***)

4. References

(1) Song, L.; Jenner, M.; Masschelein, J.; Jones, C.; Bull, M. J.; Harris, S. R.; Hartkoorn, R. C.; Vocat, A.; Romero-Canelon, I.; Coupland, P.; Webster, G.; Dunn, M.; Weiser, R.; Paisey, C.; Cole, S. T.; Parkhill, J.; Mahenthiralingam, E.; Challis, G. L. Discovery and Biosynthesis of Gladiolin: A Burkholderia Gladioli Antibiotic with Promising Activity against Mycobacterium Tuberculosis. *J. Am. Chem. Soc.* **2017**, *139*, 7974–7981.

(2) Jenner, M.; Jian, X.; Dashti, Y.; Masschelein, J.; Hobson, C.; Roberts, D. M.; Jones, C.; Harris, S.; Parkhill, J.; Raja, H. A.; Oberlies, N. H.; Pearce, C. J.; Mahenthiralingam, E.; Challis, G. L. An Unusual *Burkholderia Gladioli* Double Chain-Initiating Nonribosomal Peptide Synthetase Assembles "fungal" Icosalide Antibiotics. *Chem. Sci.* **2019**, *10* (21), 5489–5494.

(3) Baldwin, A.; Mahenthiralingam, E.; Thickett, K. M.; Honeybourne, D.; Maiden, M. C. J.; Govan, J. R.; Speert, D. P.; LiPuma, J. J.; Vandamme, P.; Dowson, C. G. Multilocus Sequence Typing Scheme That Provides Both Species and Strain Differentiation for the Burkholderia Cepacia Complex. *J. Clin. Microbiol.* **2005**, *43* (9), 4665–4673.

(4) Payne, G. W.; Vandamme, P.; Morgan, S. H.; LiPuma, J. J.; Coenye, T.; Weightman, A. J.; Jones, T. H.; Mahenthiralingam, E. Development of a recA Gene-Based Identification Approach for the Entire Burkholderia Genus. *Appl. Environ. Microbiol.* **2005**, *71* (7), 3917–3927.

(5) O'Sullivan, L. A.; Weightman, A. J.; Jones, T. H.; Marchbank, A. M.; Tiedje, J. M.; Mahenthiralingam, E. Identifying the Genetic Basis of Ecologically and Biotechnologically Useful Functions of the Bacterium Burkholderia Vietnamiensis. *Environ. Microbiol.* **2007**, *9* (4), 1017–1034.

(6) Clinical and Laboratory Standards Institute (CLSI). *Methods for Dilution Antimicrobial Susceptibility Tests for Bacteria That Grow Aerobically ; Approved Standard — Tenth Edition*. CLSI Document M07-A10. Wayne, PA: Clinical and Laboratory Standards Institute. **2012**.

(7) Clinical and Laboratory Standards Institute (CLSI). Reference Method for Broth Dilution. *Method Broth Diluition Antifung. Susceptibility Test. Yeasts; Approved Standard — Third Edition*. CLSI Document M27-A3. Wayne, PA: Clinical and Laboratory Standards Institute. **2008**.

(8) Vander Wood, D. A.; Keatinge-Clay, A. T. The Modules of Trans-Acyltransferase Assembly Lines Redefined with a Central Acyl Carrier Protein. *Proteins Struct. Funct. Bioinforma.* **2018**, *86*, 664-675.

(9) Kautsar, S. A.; Blin, K.; Shaw, S.; Navarro-Muñoz, J. C.; Muñoz, M.; Terlouw, B. R.; Van Der Hooft, J. J. J.; Van Santen, J. A.; Tracanna, V.; Suarez Duran, H. G.; V.; Andreu, V. P.; Selem-Mojica, N.; Alanjary, M.; Robinson, S. L.; Lund, G.; Epstein, S. C.; Sisto, A. C.; Charkoudian, L. K.; Jérˆ, J.; Collemare, J.; Linington, R. G.; Weber, T.; Medema, M. H. MIBiG 2.0: A Repository for Biosynthetic Gene Clusters of Known Function. *Nucleic Acids Res.* **2020**, *48*, D454–D458.

(10) Kozlov, A. M.; Darriba, D.; Flouri, T.; Morel, B.; Stamatakis, A.; Wren, J. RAxML-NG: A Fast, Scalable and User-Friendly Tool for Maximum Likelihood Phylogenetic Inference. *Bioinformatics* **2019**, *35*, 4453–4455.

(11) Larkin, M. A.; Blackshields, G.; Brown, N. P.; Chenna, R.; Mcgettigan, P. A.; McWilliam, H.; Valentin, F.; Wallace, I. M.; Wilm, A.; Lopez, R.; Thompson, J. D.; Gibson, T. J.; Higgins, D. G. Clustal W and Clustal X Version 2.0. *Bioinformatics* **2007**, *23*, 2947-2948.

(12) Zhou, S. PhD Thesis: Antibiotic Biosynthesis and Its Transcriptional Regulation in Streptomyces Bacteria. **2016**. Access: http://wrap.warwick.ac.uk/89468/.

(13) Zhou, S.; Malet, N. R.; Corre, C.; Song, L.; Challis, G. L. MmfL Catalyzes the Formation of a Butenolide Intermediate in Methylenomycin Furan Biosynthesis. *Chem. Commun.* **2020**, submitted for publication.

(14) Eble, T. E.; Bergy, M. E.; Large, C. M.; Herr, R. R.; Jackson, W. G. Isolation, Purification, and Properties of Streptovitacins A and B. *Antibiot. Annu.* **1958**, *6*, 555-559.

(15) Herr, R. R. Structures Of The Streptovitacins. *J. Am. Chem. Soc.,* **1959**, *81*, 2595-2596.

(16) Allen, M. S.; Becker, A. M.; Rickards, R. W. The Glutarimide Antibiotic 9-Methylstreptimidone: Structure, Biogenesis and Biological Activity. *Aust. J. Chem.* **1976**, *29*, 673-679.

(17) Sonoda, T.; Osada, H.; Uzawa, J.; Isono, K. Actiketal, a New Member of the Glutarimide Antibiotics. *J. Antibiot.* **1991**, *44*, 160-163.

(18) Takayasu, Y.; Tsuchiya, K.; Aoyama, T.; Sukenaga, Y. NK30424A and B, Novel Inhibitors of Lipopolysaccharide-Induced Tumour Necrosis Factor Alpha Production, Produced by Streptomyces Sp. NA30424. *J. Antibiot.* **2001**, *54*, 1111-1115.

(19) Woo, E. J.; Starks, C. M.; Carney, J. R.; Arslanian, R.; Cadapan, L.; Zavala, S.; Licari, P. Migrastatin and a New Compound, Isomigrastatin from Streptomyces Platensis. *J. Antibiot.* **2002**, *55*, 141-146.

(20) Ju, J.; Lim, S. K.; Jiang, H.; Shen, B. Migrastatin and Dorrigocins Are Shunt Metabolites of Iso-Migrastatin. *J. Am. Chem. Soc.* **2005**, *127*, 1622-1623.

(21) Ju, J.; Rajski, S. R.; Lim, S. K.; Seo, J. W.; Peters, N. R.; Hoffmann, F. M.; Shen, B. Lactimidomycin, Iso-Migrastatin and Related Glutarimide-Containing 12-Membered Macrolides Are Extremely Potent Inhibitors of Cell Migration. *J. Am. Chem. Soc.* **2009**, *131*, 1370-1371.

(22) Yin, M.; Yan, Y.; Lohman, J. R.; Huang, S. X.; Ma, M.; Zhao, G. R.; Xu, L. H.; Xiang, W.; Shen, B. Cycloheximide and Actiphenol Production in Streptomyces Sp. YIM56141 Governed by Single Biosynthetic Machinery Featuring an Acyltransferase-Less Type I Polyketide Synthase. *Org. Lett.* **2014**, *16*, 3072-3075.

(23) Zhang, D.; Yi, W.; Ge, H.; Zhang, Z.; Wu, B. Bioactive Streptoglutarimides A-J from the Marine-Derived Streptomyces Sp. ZZ741. *J. Nat. Prod.* **2019**, *82*, 2800-2808.

(24) Zhao, X. L.; Wang, H.; Xue, Z. L.; Li, J. S.; Qi, H.; Zhang, H.; Zhao, T.; Wang, J. D.; Xiang, W. S. Two New Glutarimide Antibiotics from Streptomyces sp. HS-NF-780. *J. Antibiot.* **2019**, *72,* 241-245.

(25) Lee, B.; Son, S.; Lee, J. K.; Jang, M.; Heo, K. T.; Ko, S. K.; Park, D. J.; Park, C. S.; Kim, C. J.; Ahn, J. S.; Hwang, B. Y.; Jang, J. H.; Hong, Y. S. Isolation of New Streptimidone Derivatives, Glutarimide Antibiotics from Streptomyces Sp. W3002 Using LC-MS-Guided Screening. *J. Antibiot.* **2020**, *73*, 184-188.

(26) Helfrich, E. J. N.; Ueoka, R.; Dolev, A.; Rust, M.; Meoded, R. A.; Bhushan, A.; Califano, G.; Costa, R.; Gugger, M.; Steinbeck, C.; Moreno, P.; Piel, J. Automated Structure Prediction of Trans-Acyltransferase Polyketide Synthase Products. *Nat. Chem. Biol.* **2019**, *15*, 813-821.

(27) Takemoto, Y.; Nake, K.; Kawatani, M.; Takahashi, Y.; Naganawa, H.; Imoto, M. Migrastatin, a Novel 14-Membered Ring Macrolide, Inhibits Anchorage-Independent Growth of Human Small Cell Lung Carcinoma Ms-1 Cells. *J. Antibiot.* **2001**, *54*, 1104-1107.

(28) Micoine, K.; Persich, P.; Llaveria, J.; Lam, M. H.; Maderna, A.; Loganzo, F.; Fürstner, A. Total Syntheses and Biological Reassessment of Lactimidomycin, Isomigrastatin and Congener Glutarimide Antibiotics. *Chem. Eur. J.* **2013**, *19*, 7370-7383.

(29) Larsen, B. J.; Sun, Z.; Lachacz, E.; Khomutnyk, Y.; Soellner, M. B.; Nagorny, P. Synthesis and Biological Evaluation of Lactimidomycin and Its Analogues. *Chem. Eur. J.* **2015**, *21*, 19159–67.

(30) Huang, S. X.; Yu, Z.; Robert, F.; Zhao, L. X.; Jiang, Y.; Duan, Y.; Pelletier, J.; Shen, B. Cycloheximide and Congeners as Inhibitors of Eukaryotic Protein Synthesis from Endophytic Actinomycetes Streptomyces Sps. YIM56132 and YIM56141. *J. Antibiot.* **2011**, *64*, 163-166.

(31) Sugawara, K.; NIshiyama, Y.; Toda, S.; Komiyama, N.; Hatori, M.; Moriyama, T.; Sawada, Y.; Kamei, H.; Konishi, M.; Oki, T. Lactimidomycin a New Glutarimide Group Antibiotic Production, Isolation, Structure and Biological Activity. *J. Antibiot.* **1992**, 45, 1433-1441.

(32) Chan, C.; Martin, P.; Liptrott, N. J.; Siccardi, M.; Almond, L.; Owen, A. Incompatibility of Chemical Protein Synthesis Inhibitors with Accurate Measurement of Extended Protein Degradation Rates. *Pharmacol. Res. Perspect.* **2017**, *5*, E00359.
